# Dissecting the relative contribution of ECA3 and group 8/9 cation diffusion facilitators to manganese homeostasis in *Arabidopsis thaliana*

**DOI:** 10.1002/pld3.495

**Published:** 2023-05-22

**Authors:** Emily C. Farthing, Kate C. Henbest, Tania Garcia‐Becerra, Kerry A. Peaston, Lorraine E. Williams

**Affiliations:** ^1^ School of Biological Sciences University of Southampton Southampton Hampshire UK

**Keywords:** cation diffusion facilitator (CDF), heavy metal, manganese, metal tolerance protein (MTP), natural resistance‐associated macrophage protein (NRAMP), P‐type ATPase, transport

## Abstract

Manganese (Mn) is an essential micronutrient for plant growth but becomes toxic when present in excess. A number of *Arabidopsis* proteins are involved in Mn transport including ECA3, MTPs, and NRAMPs; however, their relative contributions to Mn homeostasis remain to be demonstrated. A major focus here was to clarify the importance of ECA3 in responding to Mn deficiency and toxicity using a range of mutants. We show that ECA3 localizes to the *trans*‐Golgi and plays a major role in response to Mn deficiency with severe effects seen in *eca3 nramp1 nramp2* under low Mn supply. ECA3 plays a minor role in Mn‐toxicity tolerance, but only when the *cis*‐Golgi‐localized MTP11 is non‐functional. We also use mutants and overexpressors to determine the relative contributions of MTP members to Mn homeostasis. The *trans*‐Golgi‐localized MTP10 plays a role in Mn‐toxicity tolerance, but this is only revealed in mutants when MTP8 and MTP11 are non‐functional and when overexpressed in *mtp11* mutants. MTP8 and MTP10 confer greater Mn‐toxicity resistance to the *pmr1* yeast mutant than MTP11, and an important role for the first aspartate in the fifth transmembrane domain DxxxD motif is demonstrated. Overall, new insight into the relative influence of key transporters in Mn homeostasis is provided.

## 1 INTRODUCTION

Manganese (Mn) is an essential heavy metal micronutrient throughout all stages of plant development. The amount of Mn required in the cell hangs in a fine balance; too much can be toxic, whereas too little causes deficiency symptoms, both of which can lead to agricultural yield losses. Mn is involved in a range of critical biochemical reactions, serving structural roles or acting as a cofactor for a number of enzymes (Burnell, 1988; for review, see Williams & Pittman, 2010). Significantly, Mn plays a role in the water splitting and oxygen evolving reactions of photosynthesis as part of photosystem II (PSII; Barber, 2003, 2012), with PSII efficiency severely reduced in Mn‐deficient plants (Schmidt et al., 2016). In mitochondria, Mn superoxide dismutase (Mn‐SOD) is important for protecting against oxidative stress (Bowler et al., 1991).

Mn deficiency is one of the most frequently occurring nutritional disorders in cereal crops and is more common on sandy and calcareous soils (Jiang, 2006). Symptoms often include stunted growth, interveinal chlorosis, and slack and soft leaves, due to a reduced content of fructans and structural carbohydrates (Pearson & Rengel, 1997). Increased transpiration and decreased water‐use efficiency, associated with a decrease in epicuticular wax, have also been reported under Mn deficiency (Hebbern et al., 2009). Toxic levels of Mn can be equally detrimental to plant development, with Mn bioavailability increasing in acidic soils (Adams, 1981). Mn can replace magnesium (Mg) from key active sites to inhibit enzymatic reactions under these conditions (Bock et al., 1999). Secondary iron (Fe) deficiency can also be induced under Mn toxicity, particularly on calcareous soils where Fe availability is also limiting (Eroglu et al., 2016; Marschner, 2012). Mn toxicity symptoms include browning and cracking of roots, chlorosis of the leaf, and brown spots on mature leaves caused by accumulations of oxidized Mn and phenols, leading to losses in agricultural yield (Fecht‐Christoffers et al., 2003; Williams & Pittman, 2010).

It is therefore important to develop new and innovative approaches to target the agricultural yield losses associated with both Mn deficiency and toxicity (Williams & Pittman, 2010). Although fertilizers can be applied to help overcome Mn deficiency, their efficacy can be reduced by oxidation, and more sustainable approaches are required. There is great interest in breeding more nutrient‐efficient crops that could improve productivity on nutrient‐poor soils. Transgenic technologies also provide a wealth of opportunities to modify micronutrient efficiency and nutritional composition. Understanding the homeostatic mechanisms that control and balance levels of essential nutrients across the plant is an important starting point (Salt & Williams, 2009). Membrane transporters play a vital role in controlling metal uptake and distribution around the plant while regulating levels to avoid toxicity.

Recent years have seen great progress in the molecular characterization of transition metal transporters in *Arabidopsis*. The natural resistance‐associated macrophage protein, NRAMP1, provides high‐affinity Mn uptake at the plasma membrane (Cailliatte et al., 2010), and the Fe‐deficiency‐regulated IRT1 also has affinity for Mn uptake (Korshunova et al., 1999; Vert et al., 2002). To prevent cytoplasmic toxicity, Mn is sequestered mainly in the vacuole but has also been found in the chloroplasts, mitochondria, Golgi, and endoplasmic reticulum (ER) (Williams & Pittman, 2010). Tonoplast‐localized transporters such as Ca^2+^ and Mn^2+^/H^+^ antiporters, CAX2 and CAX5, are involved in vacuolar influx of Mn (Connorton et al., 2012; Hirschi et al., 2000) as is MTP8, an MTP (metal tolerance protein), which is also proposed to be important under Fe deficiency (Eroglu et al., 2016). MTP8 is also responsible for Mn and Fe accumulation in seeds (Chu et al., 2017; Eroglu et al., 2017). In contrast, NRAMP3 and NRAMP4 serve to redistribute Mn from the vacuole to the chloroplasts for its role in photosynthesis (Lanquar et al., 2005, 2010), whereas the *trans‐*Golgi network‐localized NRAMP2 is important under Mn deficiency (Alejandro et al., 2017; Gao et al., 2018). Recently, PAM71 (encoded by photosynthesis affected mutant 71) and CMT1 (encoded by chloroplast manganese transporter 1) have been identified as thylakoid membrane and inner envelope membrane proteins, respectively, for Mn uptake in the chloroplast (Eisenhut et al., 2018; Schneider et al., 2016; Zhang et al., 2018). PAM71 and CMT1 belong to a small five‐member protein family in *Arabidopsis*, the other members of which, PML3 (photosynthesis‐affected mutant 71 like 3) and PML4/5, are involved in Mn transport at the Golgi and endoplasmic reticulum, respectively (Hoecker et al., 2020; Yang et al., 2021).

Aside from a few examples such as NRAMP (Gao et al., 2018; Lanquar et al., 2010) and CAX transporters (Connorton et al., 2012), most of the players involved in Mn homeostasis in plants have been studied independently and often under different conditions. It is important to directly compare their relative contribution to Mn homeostasis to inform strategies for sustainable crop improvement. This study focuses on *Arabidopsis* ECA3, a P_2A_‐type ATPase (Aslam et al., 2017; Barabasz et al., 2011; Li et al., 2008; Mills et al., 2008; Pittman et al., 1999) and Group 8/9 MTP members, MTP8, MTP10, and MTP11 (Delhaize et al., 2007; Eroglu et al., 2016; Peiter et al., 2007; Ricachenevsky et al., 2013) as well as NRAMP1 and NRAMP2 (Alejandro et al., 2017; Cailliatte et al., 2010; Gao et al., 2018). Although it is clear that MTP11 is essential for alleviating Mn toxicity (Delhaize et al., 2007; Peiter et al., 2007), studies on ECA3 have indicated roles in Mn deficiency (Mills et al., 2008) but also in alleviating Mn toxicity (Li et al., 2008). To resolve this, we characterized novel *eca3 mtp11* double mutants under different Mn extremes to clarify the importance of ECA3 and MTP11 in these processes. We also address the previously disputed subcellular localization of ECA3 and MTP11 (Delhaize et al., 2007; Li et al., 2008; Mills et al., 2008; Peiter et al., 2007). Further, we assessed the contribution of the Mn‐MTPs in Mn homeostasis, generating and directly comparing novel double and triple mutants for *mtp8*, *mtp10*, and *mtp11*. From this and localization studies, a role for MTP10 at the Golgi in alleviating Mn toxicity was identified. Additionally, although NRAMP1 and NRAMP2 are known to play a role in Mn deficiency (Cailliatte et al., 2010; Gao et al., 2018), here the relative importance of each NRAMP and ECA3 was investigated by comparing single, double, and triple *nramp1*, *nramp2*, and *eca3* mutants under Mn‐deficiency conditions. This study provides further understanding of key transporters and their relative importance in contributing to Mn homeostasis under low and high Mn.

## 2 RESULTS

A specific aim of this study was to clarify the importance of ECA3 under Mn deficiency and toxicity, as it is currently unclear where it has its major contribution. The first part of the study investigates its role under Mn deficiency. NRAMP1 and NRAMP2 transporters have important roles in Mn deficiency, and so a comparison of mutants was undertaken to investigate the relative effects when ECA3, NRAMP1, and NRAMP2 were knocked out.

### 2.1 The relative contributions of ECA3, NRAMP1, and NRAMP2 under Mn deficiency

**FIGURE 1:**
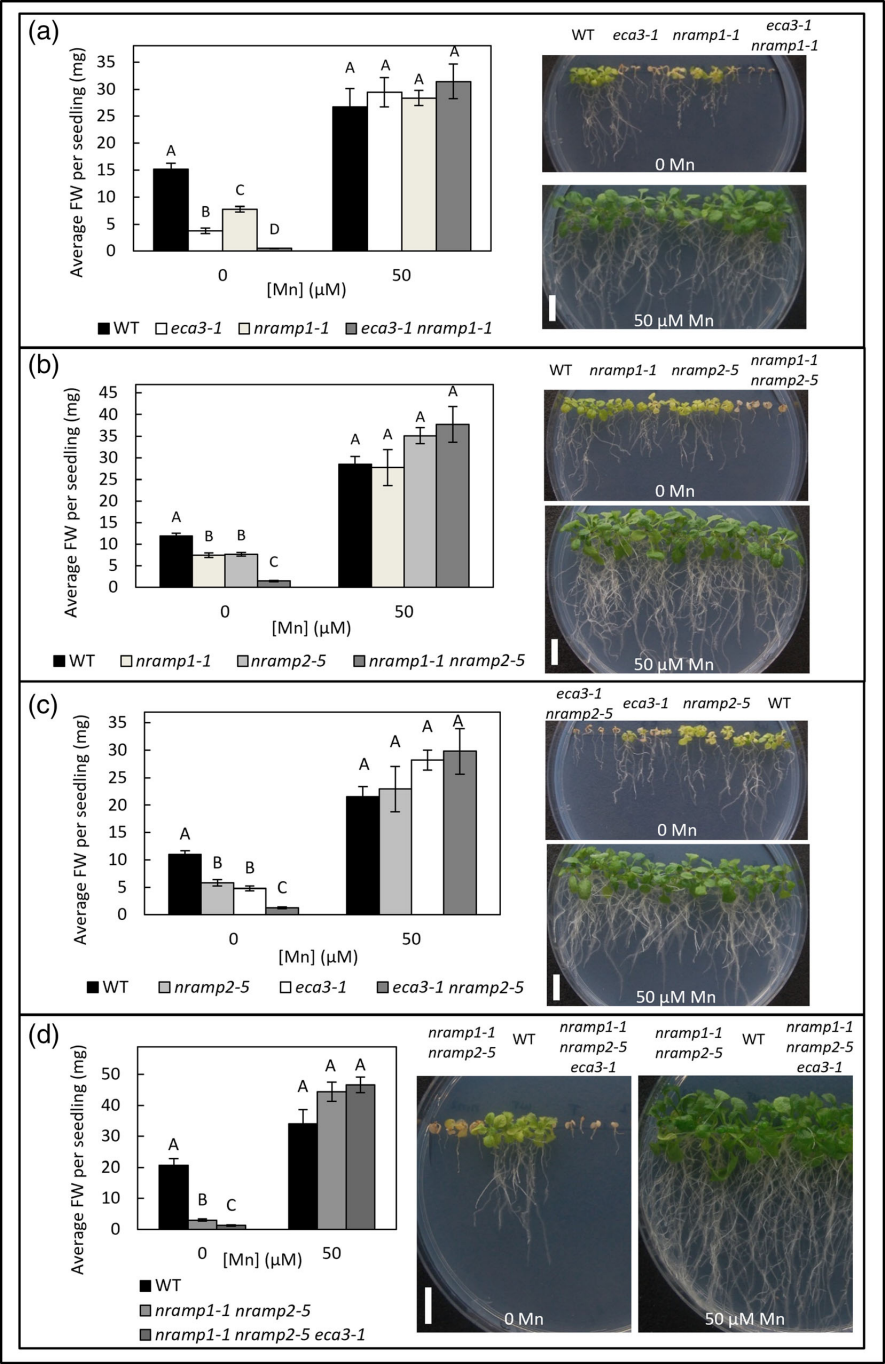
Comparison of *nramp1‐1*, *nramp2‐5*, *eca3‐1* single, double, and triple mutants under Mn deficiency. Comparison of Columbia 8 wild type (WT) with single mutants of *eca3‐1*, *nramp1‐1*, and *nramp2‐5* and corresponding double mutants (a) *eca3‐1 nramp1‐1*, (b) *nramp1‐1 nramp2‐5*, (c) *eca3‐1 nramp2‐1*, and (d) *nramp1‐1 nramp2‐5 eca3‐1* when grown for 21 days on ½ MS supplemented with either 0 or 50 μM MnSO_4_. Data show mean fresh weight (FW) per seedling (±SE) calculated for six plates per condition, with four seedlings per genotype per plate. Statistical significance was assessed with two‐way ANOVA and Tukey's post hoc test. Means not sharing a letter at each individual concentration are significantly different. Photographs display representative plant growth at basal (50 μM) and deficient (0 μM) Mn concentrations. White bar = 1 cm.

The first part of this study investigates ECA3, NRAMP1, and NRAMP2 by comparing single mutants and generating double and triple mutants to determine their response under Mn‐deficient conditions. T‐DNA insertion mutants *eca3‐1* (Mills et al., 2008), *nramp1‐1* (Cailliatte et al., 2010), and *nramp2‐5* were crossed to isolate homozygous double mutants *eca3‐1 nramp1‐1*, *eca3‐1 nramp2‐5*, and *nramp1‐1 nramp2‐5*. A triple mutant was then generated from crosses of double mutants for *eca3‐1 nramp2‐5* and *nramp1‐1 nramp2‐5*. RT‐PCR was used to confirm double and triple mutants at the RNA level (Figure S1). When mutants were grown on half‐strength MS medium (1/2 MS) with Mn omitted or supplied at basal levels (50 μM Mn), both *nramp1‐1* and *nramp2‐5* showed stunted growth under Mn deficiency, whereas *eca3‐1* showed even greater sensitivity (stunting and chlorosis) (Figure 1). Each of the double mutants, *eca3‐1 nramp1‐1*, *eca3‐1 nramp2‐5*, and *nramp1‐1 nramp2‐5* displayed additive sensitivities under Mn deficiency compared to the corresponding single mutants. When grown alongside the *nramp1‐1 nramp2‐5* double mutant, the *nramp1‐1 nramp2‐5 eca3‐1* triple mutant showed further sensitivity to Mn deficiency (Figure 1d). This indicates distinct contributions of these transporters to Mn deficiency.

### 2.2 Mn‐sensitivity of *eca3 mtp11* double mutants under two Ca regimes

MTP11 has a major role in mitigating Mn toxicity (Delhaize et al., 2007; Peiter et al., 2007), but the role of ECA3 is less certain. Therefore, it is important to compare their relative contributions more directly. Double mutants were generated and compared to single mutants to investigate their response under different Mn conditions. T‐DNA insertion mutants *eca3‐1* and *eca3‐2* (Mills et al., 2008) were both crossed with *mtp11‐1* (Delhaize et al., 2007), and homozygous double mutants, *eca3‐1 mtp11‐1* and *eca3‐2 mtp11‐1*, were isolated. RT‐PCR was used to confirm double mutants at the RNA level (Figure S2A).

**FIGURE 2:**
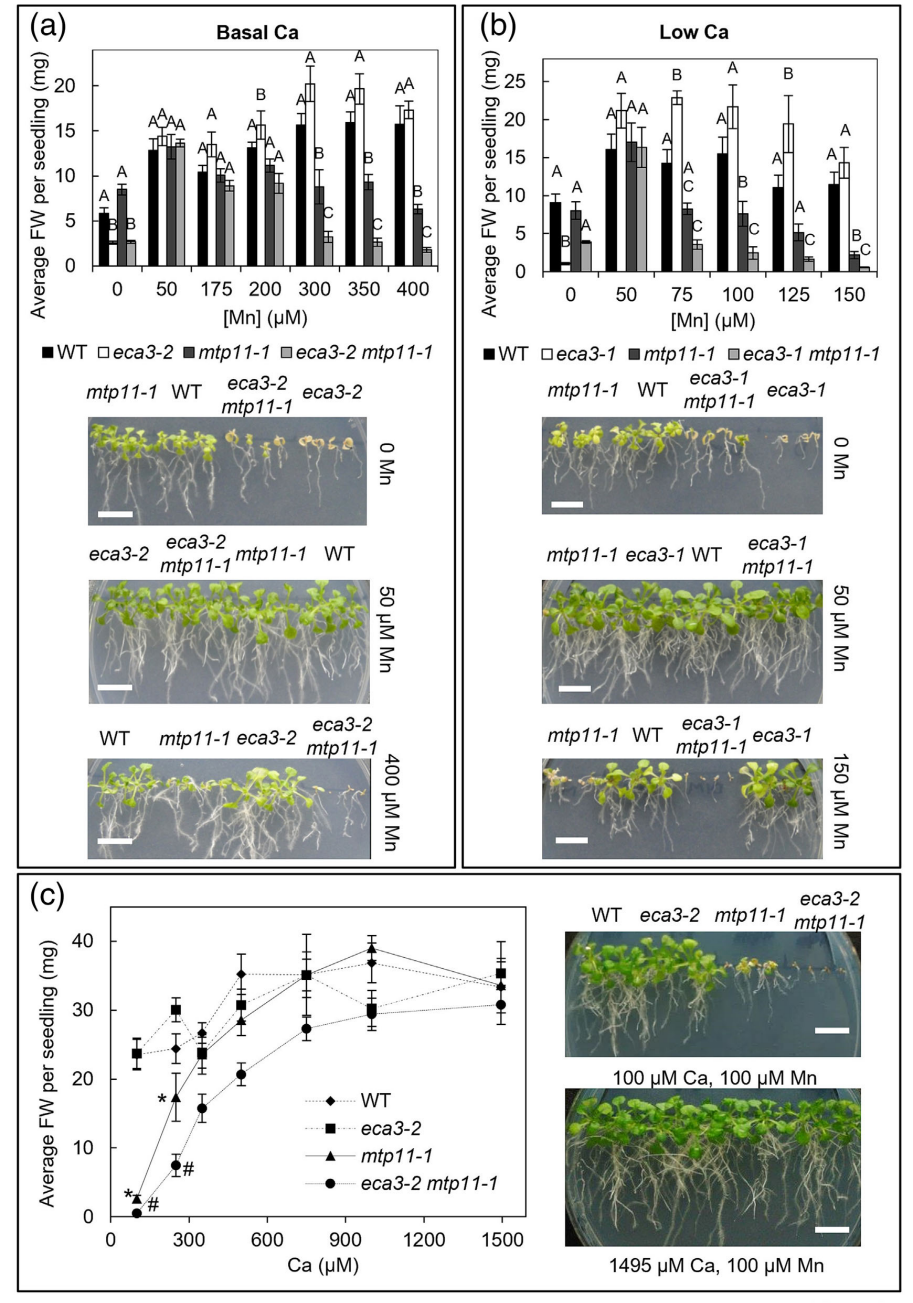
*eca3 mtp11* double mutants show increased susceptibility to Mn toxicity when grown under both Ca regimes. (a,b) Comparison of Col8 WT, *mtp11‐1* and either (a) *eca3‐2* and *eca3‐2 mtp11‐1* or (b) *eca3‐1* and *eca3‐1 mtp11‐1* under two Ca regimes. Plants were grown for 19 days on ½ MS supplemented with a range of MnSO_4_ concentrations, and either (a) 1495 μM Ca (basal Ca) or (b) 100 μM Ca (low Ca). (c) Increasing Ca alleviates Mn toxicity. Plants were grown on ½ MS containing 100 μM MnSO_4_ with a range of CaCl_2_ concentrations for 20 days. Data show mean FW (mg) per seedling (±SE) calculated for six plates, with four seedlings per genotype per plate. Statistical significance was assessed with two‐way ANOVA and Tukey's post hoc test. (a,b) Means not sharing a letter at a particular condition are significantly different. (c) *, significantly smaller than WT; #, significantly smaller than *mtp11‐1*. Photographs display representative growth across different conditions. White scale bar = 1 cm. See also Figures S2 and S3 and Table S1.

Mutants were compared across a range of Mn conditions from deficiency (0 Mn supplied) to excess Mn to clarify the role of ECA3 and MTP11 in Mn homeostasis (Figure 2). Because ECA3 is proposed to transport Ca as well as Mn, and *eca3* mutants were previously shown to be extremely stunted on low‐Ca and low‐Mn medium (Mills et al., 2008), we employed two calcium (Ca) regimes. Mutants were grown on half‐strength MS medium (1/2 MS) at standard Ca (1.495mM) and low Ca (100 μM). All mutants grew similarly to wild‐type (WT) plants under basal Mn conditions (50 μM Mn). Under Mn‐deficiency conditions (0 μM Mn) at both Ca levels, whereas *eca3‐1* and *eca3‐2* single mutants displayed hypersensitivity, *mtp11‐1* performed similarly to WT. Under these conditions, the *eca3 mtp11* double mutants performed similarly or even better than the *eca3* single mutants (Figure 2a,b). A further mutant, *eca3‐4*, was also sensitive to Mn deficiency (stunting and chlorosis), supporting findings by Mills et al. (2008) that ECA3 has a crucial role in Mn nutrition. Under elevated Mn, *mtp11‐1* displayed a hypersensitive phenotype (stunting and chlorosis), which was apparent at lower Mn concentrations under the low Ca regime (100 μM Mn compared with 300 μM Mn). Importantly, the *eca3‐2 mtp11‐1* and *eca3‐1 mtp11‐1* double mutants displayed a more severe sensitivity to Mn toxicity than *mtp11‐1* under both standard and low Ca (Figure 2a,b). At elevated Mn, *eca3‐1* and *eca3‐2* single mutants generally responded similarly to WT. In a separate experiment, *eca3‐4* also showed no sensitivity to Mn toxicity under either Ca regime (Figure S3), contradicting reports by Li et al. (2008) that showed *eca3‐4* displaying inhibition at just 50 μM Mn. Therefore, a role in alleviating toxicity for ECA3 is only apparent when MTP11 is non‐functional.

It was noticeable that the Mn‐toxicity‐dependent phenotype of *mtp11* and *eca3 mtp11* mutants is observed at lower Mn concentrations under the low Ca regime, than under the basal Ca regime (Figure 2a,b). To further investigate this Mn/Ca antagonism, Mn was provided as 100 μM, with Ca ranging from 100 to 1495 μM (Figure 2c). Our results show that 100 μM Mn becomes increasingly inhibitory to sensitive genotypes (*mtp11‐1* and *eca3‐2 mtp11‐1*) when Ca is lowered, with significant stunting compared with WT below 300 μM Ca. At this lower range, the *eca3‐2 mtp11‐1* double mutant is significantly more inhibited than the *mtp11‐1* single mutant.

### 2.3 ECA3 and MTP11 target different Golgi compartments

**FIGURE 3:**
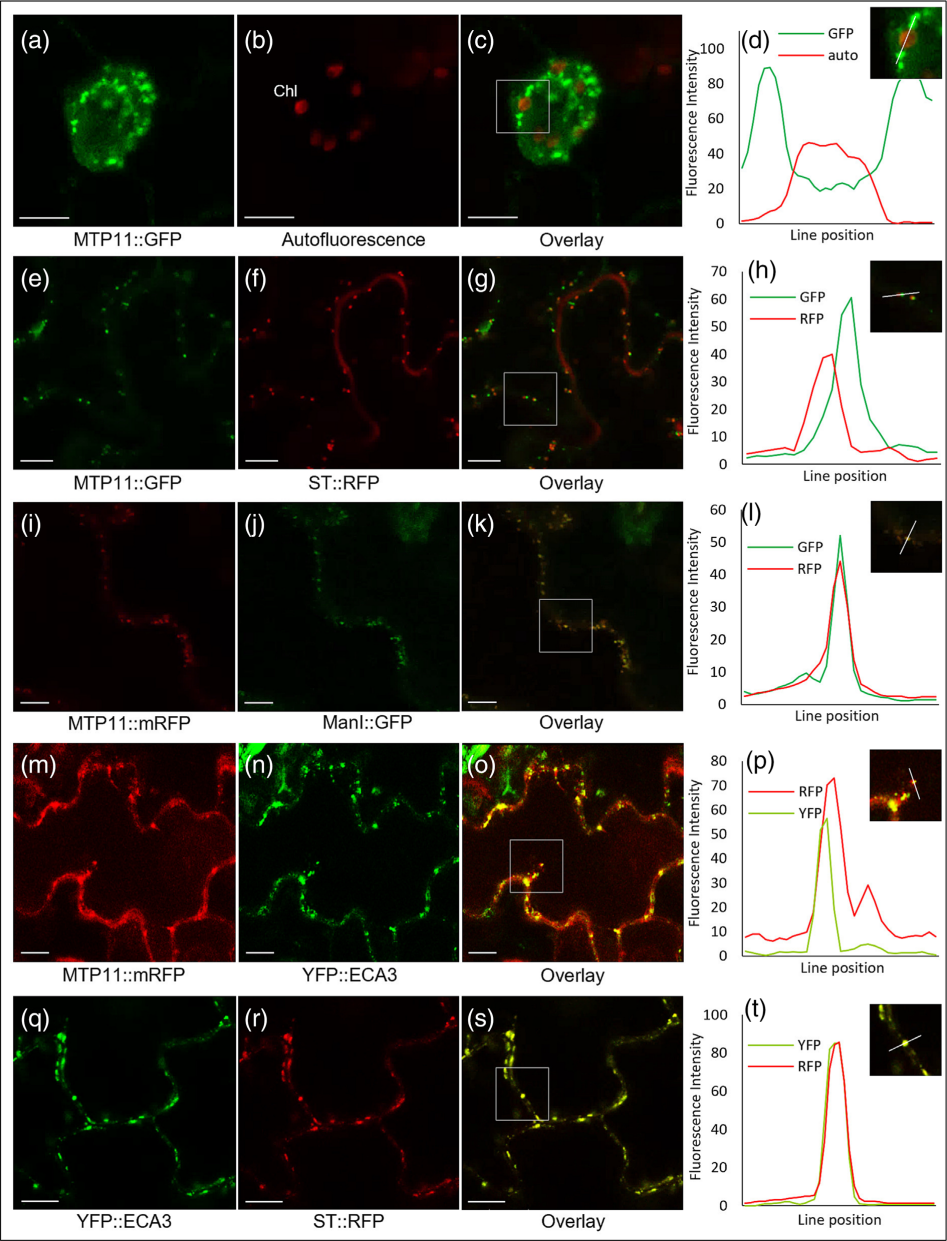
MTP11 and ECA3 target the *cis*‐Golgi and *trans*‐Golgi *in planta*, respectively. (a) Guard cell of 7‐day‐old *Arabidopsis* seedling stably expressing MTP11::GFP; (b) chlorophyll autofluorescence (Chl); (c) merged image of (a) and (b); (d) fluorescence intensity profiles of GFP and chlorophyll autofluorescence were measured along a line spanning, magnified from the inset shown in (c). (e) Transient expression of MTP11::GFP in tobacco epidermal cells, co‐expressed with (f) *trans*‐Golgi‐marker sialyl transferase (ST::RFP); (g) merged image of (e) and (f); (h) fluorescence intensity profiles of GFP and RFP showing that MTP11 and ST sit adjacent to each other but do not entirely overlap. (i) Fluorescent signal of MTP11::mRFP in tobacco epidermal cells, co‐expressed with (j) *cis*‐Golgi‐marker ManI::GFP; (k) merged image of (i) and (k); (l) overlapping fluorescence intensity profiles showing that MTP11::GFP and ManI::GFP colocalize. (m) Transient expression of MTP11::mRFP in tobacco epidermal cells, co‐expressed with (n) YFP::ECA3; (o) merged image of (m) and (n); (p) partial but incomplete fluorescence intensity overlap for MTP11::RFP and YFP::ECA3 signals. (q) Transient expression of YFP::ECA3 in tobacco epidermal cells, co‐expressed with (r) *trans*‐Golgi‐marker ST::RFP; (s) merged image of (q) and (r); (t) fluorescence intensity profiles show colocalization of YFP::ECA3 and ST::RFP. Confocal micrographs are representative images; white scale bar = 10 μm.

Although the role of MTP11 in contributing to Mn detoxification is clear, its localization is uncertain and has been reported to target either the *trans‐*Golgi network (Peiter et al., 2007) or the pre‐vacuolar compartment (PVC; Delhaize et al., 2007). Similarly, ECA3 has been proposed to target the Golgi (Mills et al., 2008) or the PVC or another endosomal compartment (Li et al., 2008). Here when stably expressed in *Arabidopsis*, we show that MTP11 displays a punctate expression pattern (Figure 3a–d). Further, transient expression in tobacco shows partial overlap with *trans‐*Golgi marker sialyl transferase (ST)::RFP and strong overlap with *cis‐*Golgi marker, ManI::GFP (Figure 3e–l). When compared directly, ECA3 and MTP11 show areas of distinct but incomplete overlap (Figure 3m–p); correspondingly, ECA3 displays strong overlap with *trans‐*Golgi marker ST::RFP (Figure 3q–t). It appears, therefore, that ECA3 targets the *trans‐*Golgi, whereas MTP11 targets the *cis‐*Golgi.

### 2.4 Determining the contribution of Group 8/9 MTPs to Mn homeostasis

The second key aim of this study was to determine the relative contribution of the *Arabidopsis* Mn‐MTPs in Mn homeostasis. An updated phylogenetic tree for putative plant Mn‐MTPs is presented in Figure S4. This analysis includes proteins that have not been reported previously: members from *Brassica rapa*, identified from Phytozome9.1, and the putative *Hordeum vulgare* (barley) HvMTP11, identified by searching the International Barley Sequencing Consortium (Table S2). Phylogenetic analysis confirms the clustering of MTP8 proteins in Group 8, separately from those in Group 9 (MTP9, MTP10, and MTP11). It appears that most monocots, including rice, barley, and sorghum, possess two MTP8 Group 8 members, whereas *Arabidopsis* possesses only one. *Arabidopsis* contains three Group 9 MTPs: MTP11 and more divergent MTP9 and MTP10. Monocots, meanwhile, contain two MTP11 sequences but only one other MTP9. All proteins clustering into Group 8 and Group 9 are confirmed to carry the MTP signature sequence and two DxxxD domains on putative transmembrane domains (TMDs) two and five, characteristic of the Mn‐MTPs (Montanini et al., 2007). *Arabidopsis* MTP1, MTP6, and MTP7 are included in the phylogenetic analysis, clearly clustering separately into Groups 1, 6, and 7, respectively (Gustin et al., 2011).

The cDNAs were amplified and cloned for functional analysis of the *Arabidopsis* Group 8/9 MTPs: *MTP8*, *MTP9*, *MTP10*, and *MTP11*. Sequences for *MTP8* and *MTP10* are identical to information listed on The Arabidopsis Information Resource (TAIR), whereas *MTP11* corresponds to that previously published (Delhaize et al., 2007; Peiter et al., 2007). Two gene models are listed on TAIR for the *MTP9* coding sequence, differing in 36 bases. The cDNA cloned in this study corresponded to the *MTP9.1* model, here referred to simply as *MTP9*. The corresponding intron/exon structures are shown in Figure S5. Six TMDs are predicted for each of MTP8, MTP9, MTP10, and MTP11 when using the consensus output from AramTmConsens on ARAMEMNON (Schwacke et al., 2003), a database that integrates outputs of 18 helix prediction programs. Although an extra TMD is listed towards the 5′ end of MTP10 by AramTmConsensus, the consensus score is very low (0.19), below the consensus threshold for a TMD of 0.42. The predicted six TMDs are labeled in the alignment of MTP8, MTP9, MTP10, and MTP11 in Figure S6. Also labeled are the DxxxD domains of TMDs two and five, which are substituted for HxxxD in zinc‐transporting MTP1 (Montanini et al., 2007).

### 2.5 Generation and analysis of Group MTP8/9 mutants

To determine the contribution to Mn homeostasis, we isolated single T‐DNA insertion mutants for MTP8 (*mtp8‐1* and *mtp8‐2* in the Columbia background; described in Eroglu et al., 2016) and MTP10 (*mtp10‐1* and *mtp10‐2*, in the Columbia background). Confirmed insertion sites are highlighted on the intron/exon diagram structures in Figure S5. We also generated a series of double and triple mutants for *mtp8*, *mtp10*, and *mtp11*; confirmation of these mutants is shown in Figure S2B–E. At the time, there was no *mtp9* mutant available in the same background.

**FIGURE 4:**
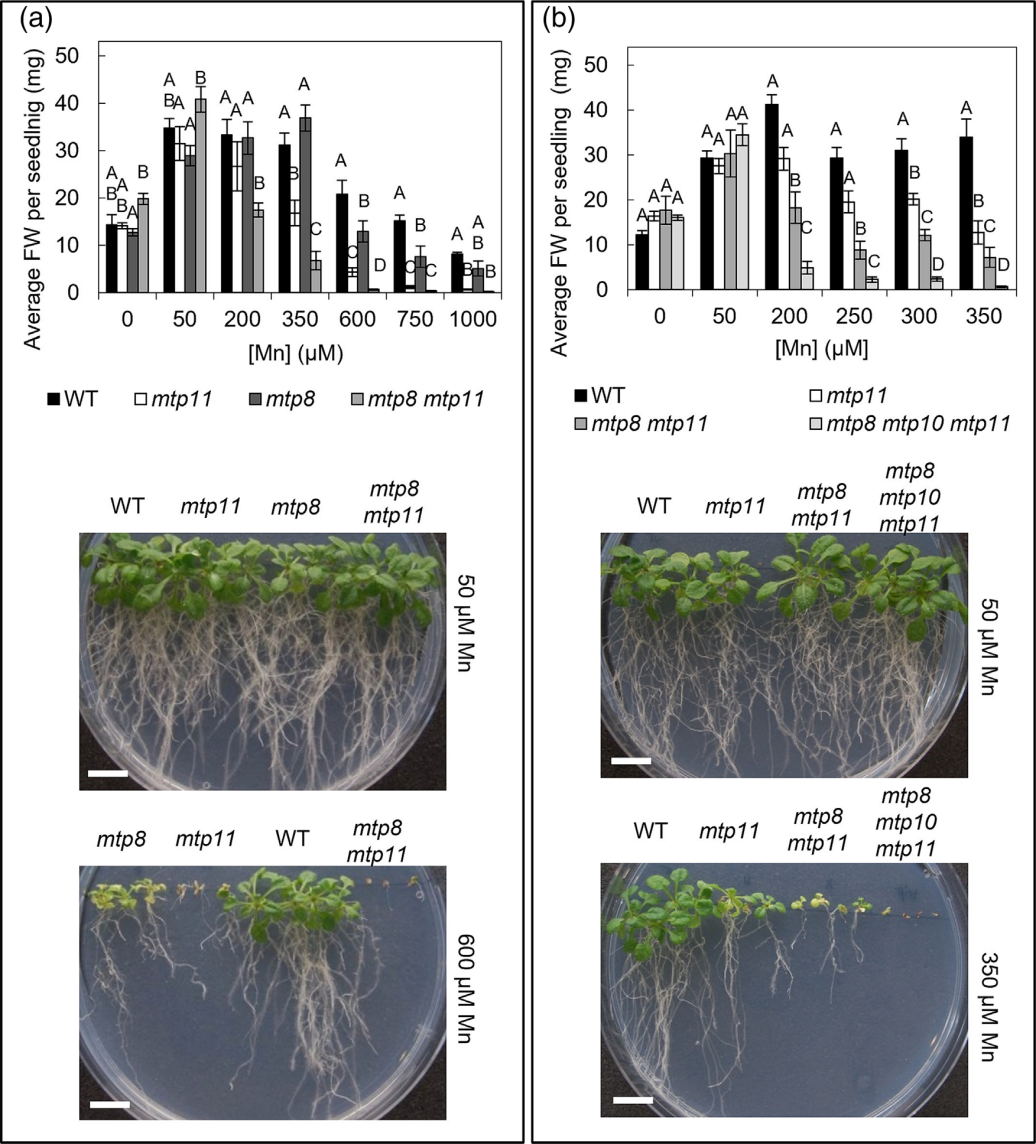
Increased susceptibility to Mn toxicity of *mtp8 mtp11* double mutant and *mtp8 mtp10 mtp11* triple mutant under basal Ca conditions. Average fresh weight (FW) per seedling of Col8 WT with either (a) *mtp11‐1*, *mtp8‐2*, and *mtp8‐2 mtp11‐1* or (b) *mtp11‐1*, *mtp8‐2 mtp11‐1*, and *mtp8‐2 mtp10‐1 mtp11‐1*. Plants were grown on ½ MS supplemented with a range of MnSO_4_ concentrations for 21 days. Data show mean FW (mg) per seedling (±SE) calculated for six plates, with four seedlings per genotype per plate. Statistical significance was assessed with two‐way ANOVA and Tukey's post hoc test. Means not sharing a letter at each individual concentration are significant. Photographs display representative plant growth across different Mn concentrations. White bar = 1 cm. See also Figures S4–S7.

When tested under Mn deficiency, none of the single mutants were significantly affected compared with WT (Figure S7). A direct comparison for *mtp8‐2*, *mtp11‐1*, and the corresponding double mutant is shown, across a range of Mn treatments, under both basal Ca (Figure 4) and low Ca (Figure S7A,B). Both single mutants were inhibited by Mn toxicity; *mtp11‐1* was more susceptible than *mtp8‐2*, becoming hypersensitive at lower concentrations and displaying greater levels of stunting and chlorosis. Sensitivity to elevated Mn was exacerbated by low Ca conditions. The *mtp8‐2 mtp11‐1* double mutant displayed greater sensitivity than either of the singles under both Ca regimes. Also, under low Ca conditions, the germination of *mtp8‐2 mtp11‐1* dropped from 100% at 50 μM Mn to 4% at 150 μM Mn, whereas the germination of the other genotypes remained unaffected (Figure S8).

**FIGURE 5:**
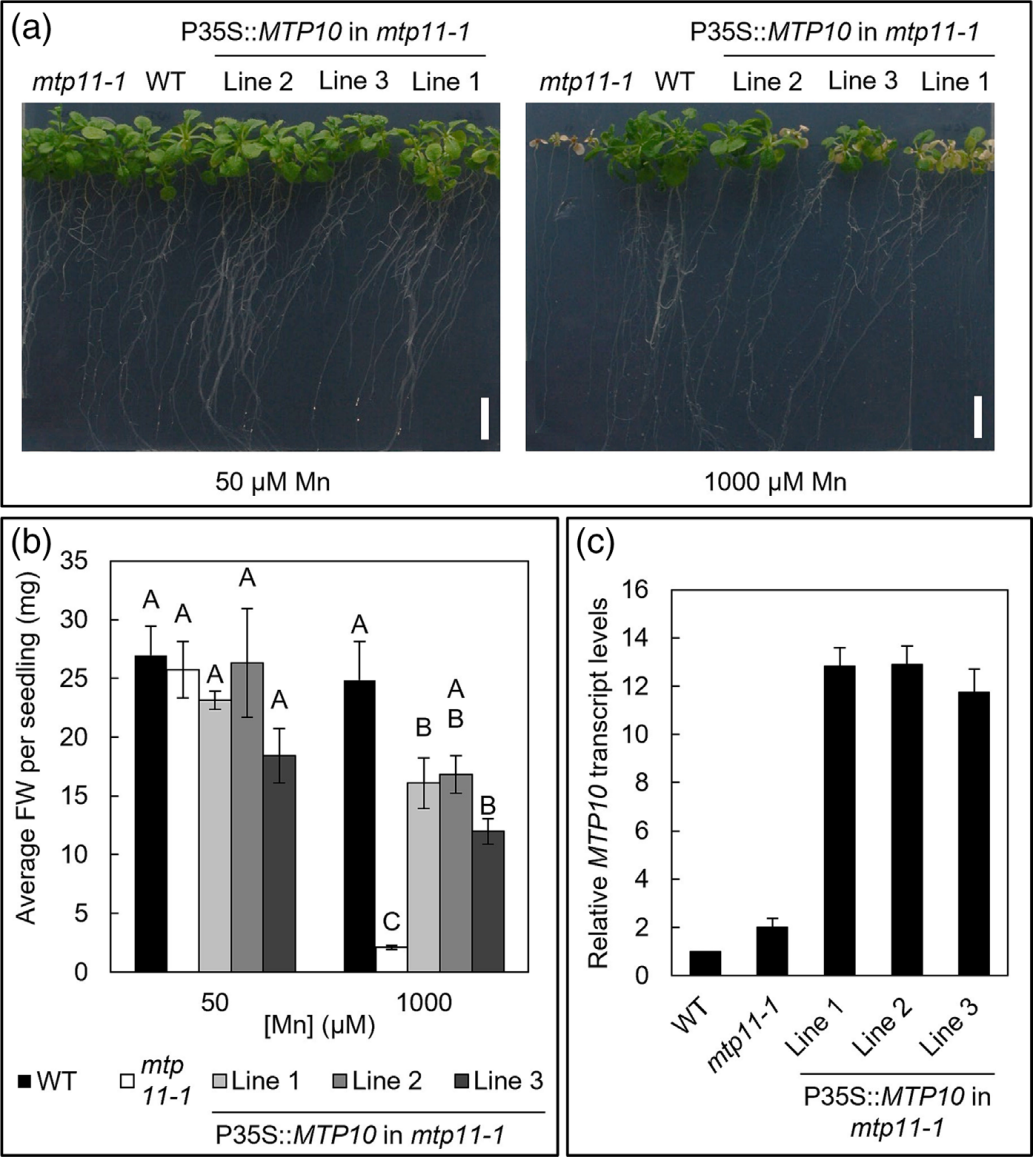
Overexpression of MTP10 in *mtp11‐1* rescues the Mn toxicity susceptibility phenotype of the mutant. (a) Representative plant growth of Col8 WT, *mtp11‐1* and three independent *mtp11‐1* lines expressing *Pro35S::MTP10*, after 21‐day growth on ½ MS containing basal Ca levels (1495 μM Ca), under basal (50 μM) and toxic (1000 μM) Mn conditions. (b) Average fresh weight (FW) per seedling. Data show mean FW (mg) per seedling (±SE) calculated for six plates, with four seedlings per genotype per plate. Statistical significance was assessed with two‐way ANOVA and Tukey's post hoc test. Means not sharing a letter at a particular concentration are significantly different. (c) RT‐qPCR shows three transformed *mtp11‐1* lines express *MTP10* at higher levels than WT.

The role of MTP10 in Mn homeostasis has not previously been reported. Knocking out MTP10 in addition to *mtp11*, in the *mtp10 mtp11* double mutants, had no additional effect compared to growth of the single *mtp11* mutant (Figure S7C). Although the average FW for the *mtp10 mtp11* mutant was consistently lower than *mtp11* under Mn toxicity, this was not significant. Similarly, knocking out MTP10 in addition to *mtp8*, in the *mtp8 mtp10* double mutant, had no additional effect compared the single *mtp8* mutant (Figure S7D). However, an underlying contribution of MTP10 to alleviating Mn toxicity did become apparent in the triple mutant, *mtp8‐2 mtp10‐1 mtp11‐1*, which was significantly more inhibited by Mn toxicity than the double *mtp8‐2 mtp11‐1*, under both Ca regimes (Figures 4b and S7B). The triple mutant was inhibited at 200 μM Mn under basal Ca conditions (Figure 4b), although 10 μM Mn was sufficient to induce a toxicity phenotype under low Ca (Figure S7B). Direct comparison of these mutants reveals a role for MTP10 in alleviating Mn toxicity, but this is only observable when MTP8 and MTP11 are non‐functional. The potential contribution of MTP10 to Mn homeostasis is further observed when expressed in the *mtp11* single mutant; under Mn toxic conditions, MTP10 was able to partially restore the growth of the sensitive *mtp11* mutant (Figure 5). Multiple independent lines showed a rescuing of the chlorotic phenotype and increased lateral root growth in comparison with the mutant (Figure 5a).

**FIGURE 6:**
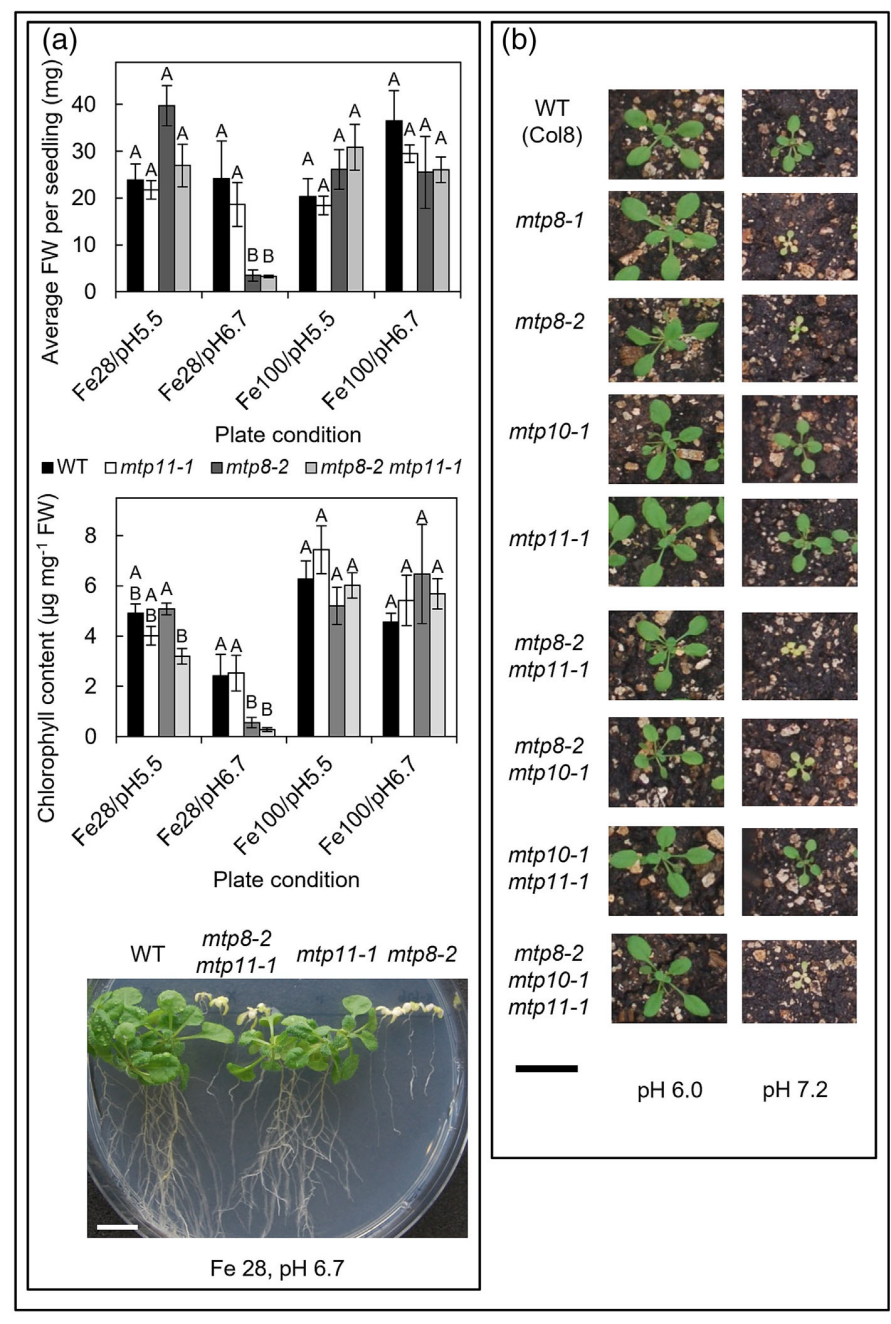
MTP8 is the primary Mn‐MTP involved in alleviating symptoms under low Fe/high pH conditions. (a) Average fresh weight (FW; mg) per seedling and average chlorophyll (chl; μg) per mg FW of Col8 WT, *mtp11‐1*, *mtp8‐2,* and *mtp8‐2 mtp11‐1*. Plants were grown for 23 days on ½ MS (modified Eroglu regime) supplemented with either 28 μM or 100 μM FeNaEDTA (Fe28 and Fe100, respectively), buffered to pH 5.5 or 6.7 with NaOH. Data show mean FW (mg) per seedling (±SE) calculated for six plates, with four seedlings per genotype per plate. Statistical significance was assessed with MANOVA and Tukey's post hoc test. Means not sharing a letter at a particular condition are significantly different. Photograph displays plant growth under 28 μM Fe, buffered to pH 6.7. White scale bar = 1 cm. (b) Comparison of different MTP single, double, and triple mutants on non‐limed (pH 6.0) and limed (pH 7.2) soil. Plants photographed after 19‐day growth. Photos are representative of 24 plants per genotype under each condition. Black scale bar = 2 cm. See also Figures S6 and S7 and Movie S1.

An important interaction in Mn homeostasis is the antagonism between Mn and Fe, and it has been proposed that MTP8 functions at the tonoplast to sequester Mn into the vacuole in order to prevent Mn‐induced inhibition of the Fe‐deficiency response machinery (Eroglu et al., 2016). A stunted, chlorotic phenotype was seen for *mtp8‐1* and *mtp8‐2* under low Fe availability induced by high pH (½ MS plates with 28 μM Fe pH 6.7; Eroglu et al., 2016). In order to determine whether other MTP members are involved in this proposed mechanism, *mtp8‐2*, *mtp11‐1*, single and double mutants were grown on plates under the same conditions (Figure 6a), whereas further *mtp* single, double, and triple mutants were grown on limed soil where pH was raised to 7.2 (Figure 6b). In both experiments, plants with *MTP8* mutations were stunted and chlorotic. The other single mutants were unaffected compared with WT, and there was not a marked difference in double and triple mutants with a non‐functional MTP8 to the *mtp8‐2* single mutant (Figure 6), indicating that only MTP8 plays the major role under these conditions.

### 2.6 MTP8 targets the tonoplast *in planta* whereas MTP10 is Golgi‐localized

**FIGURE 7:**
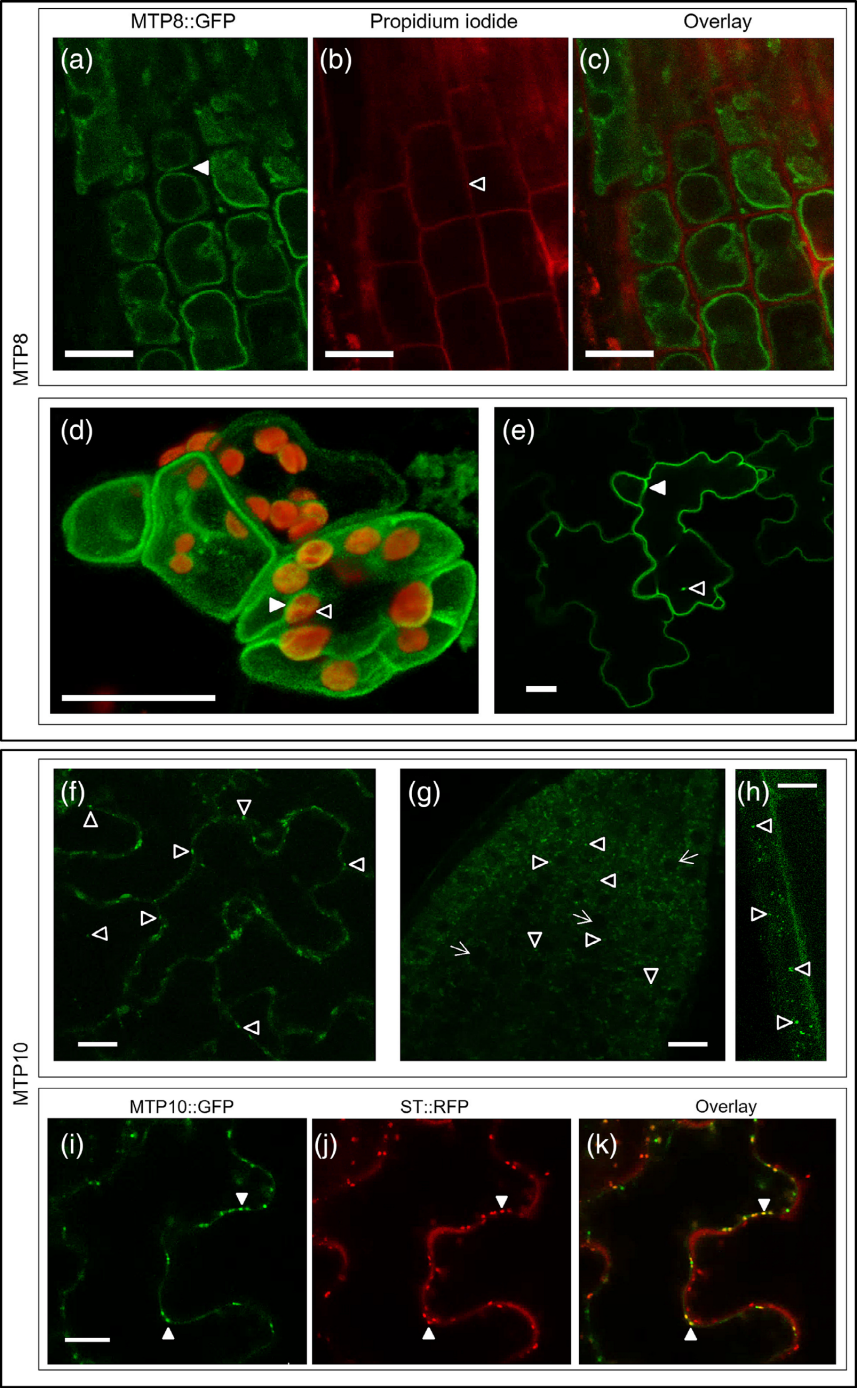
MTP8 and MTP10 target the tonoplast and *trans*‐Golgi *in planta*, respectively. (a–d) MTP8::GFP targets the tonoplast when stably expressed in 7‐day‐old Arabidopsis seedlings. (a) MTP8::GFP in root cells (green signal; filled arrow highlights double vacuole) with (b) cell walls stained with propidium iodide (red signal; unfilled arrow); (c) merged image of (a) and (b). (d) Merged 3D Z‐stack of guard cell and neighboring epidermal cells, with MTP8::GFP (green; unfilled arrow) and chloroplast autofluorescence (red signal; filled arrow). MTP8 signal does not enclose the chloroplast, which is characteristic of tonoplast localization. (e) MTP8::GFP shows tonoplast localization when transiently expressed in tobacco epidermal cells. Formation of two transvacuolar strands is indicated (filled and unfilled arrows). (f–k) MTP10::GFP targets the *trans*‐Golgi. Stable expression of MTP10::GFP in 7‐day‐old *Arabidopsis* seedlings, displaying punctate fluorescent expression in (f) epidermal leaf cells and (g,h) root cells. Punctate signal is clearly distinct from developing vacuoles, marked with small white arrow. (i) Transient expression of MTP10::GFP in tobacco epidermal cells (green signal), colocalizing with *trans*‐Golgi marker sialyl transferase (ST::RFP; red signal). (k) Merged image of (i) and (j); filled arrows indicate overlapping signal. White bar = 10 μm. See also Movie S1.

Previously, MTP8 has been localized to the tonoplast when expressed in mesophyll protoplasts (Eroglu et al., 2016) and *Nicotiana benthamiana* leaves (Zhang et al., 2021). Here, we take a step forward and show that MTP8 targets the tonoplast in both root and shoot cells when stably expressed in *Arabidopsis*. MTP8::GFP is clearly visible at the developing vacuolar membranes in many cells of the root (Figure 7a–c) and is clearly internal to chloroplast autofluorescence in stomatal guard cells (Figure 7d). Expression of MTP8::GFP is also characteristic of the tonoplast when transiently expressed in tobacco epidermal cells (Figure 7e); Movie S1 shows formation of transvacuolar strands (TVS) and TVS trafficking across the vacuole. Contrastingly, MTP10::GFP is more punctate in appearance when expressed stably in *Arabidopsis* (Figure 7f–h). MTP10::GFP expression overlaps with ST::RFP when transiently expressed in tobacco (Figure 7i–k), suggesting MTP10 targets the *trans‐*Golgi.

**FIGURE 8:**
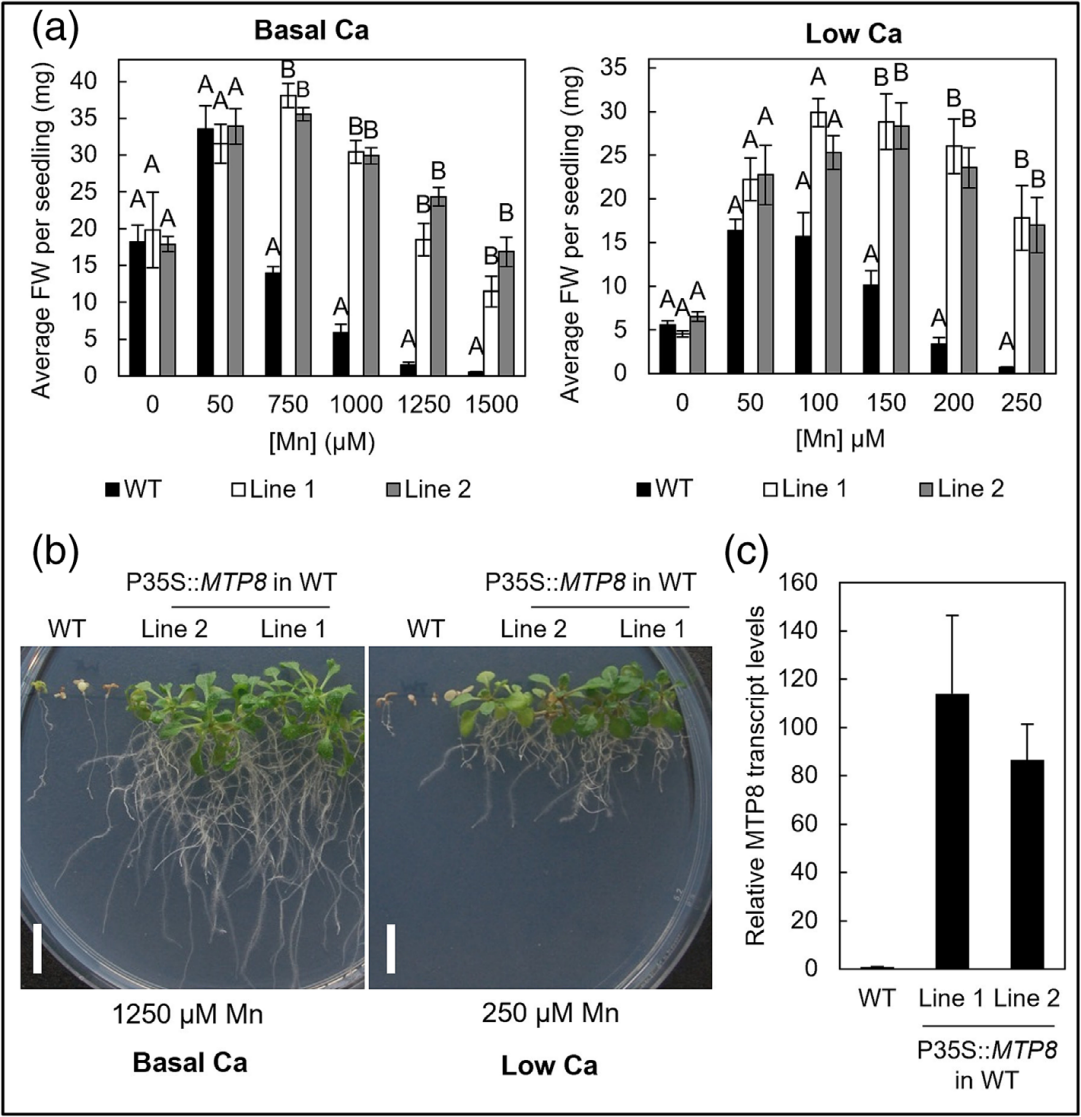
Overexpression of MTP8 in WT *Arabidopsis* confers improved tolerance to excess Mn under both Ca regimes. (a) Average fresh weight (FW) per seedling of Col8 WT, *mtp8‐2*, and two independent WT lines expressing Pro35S::MTP8, after 21‐day growth on ½ MS containing basal Ca levels (1495 μM Ca) or 24‐day growth on ½ MS containing low Ca levels (100 μM Mn). Data show mean FW (mg) per seedling (±SE) calculated for six plates, with four seedlings per genotype per plate. Statistical significance was assessed with two‐way ANOVA and Tukey's post hoc test. Means not sharing a letter at a particular concentration are significantly different. (b) Photographs display representative plant growth under different Mn toxicity. White bar = 1 cm. (c) RTqPCR shows two transformed WT lines express MTP8 at higher levels than WT.

We generated WT lines transformed with *Pro35S::MTP8* (Figure 8). Overexpressing lines displayed significantly enhanced resistance to elevated Mn under both Ca regimes (Figure 8a,b). Overexpressing lines have previously shown altered Mn accumulation (Eroglu et al., 2016) and Chu et al. (2017) showed decreased sensitivity to moderate Mn toxicity in a single overexpressing line. Here we show, in two independent MTP8‐overexpressing lines, that this hypertolerant growth phenotype is present even under extreme Mn‐toxicity conditions.

### 2.7 Direct comparison of MTP8, MTP10, and MTP11 when heterologously expressed in yeast

**FIGURE 9:**
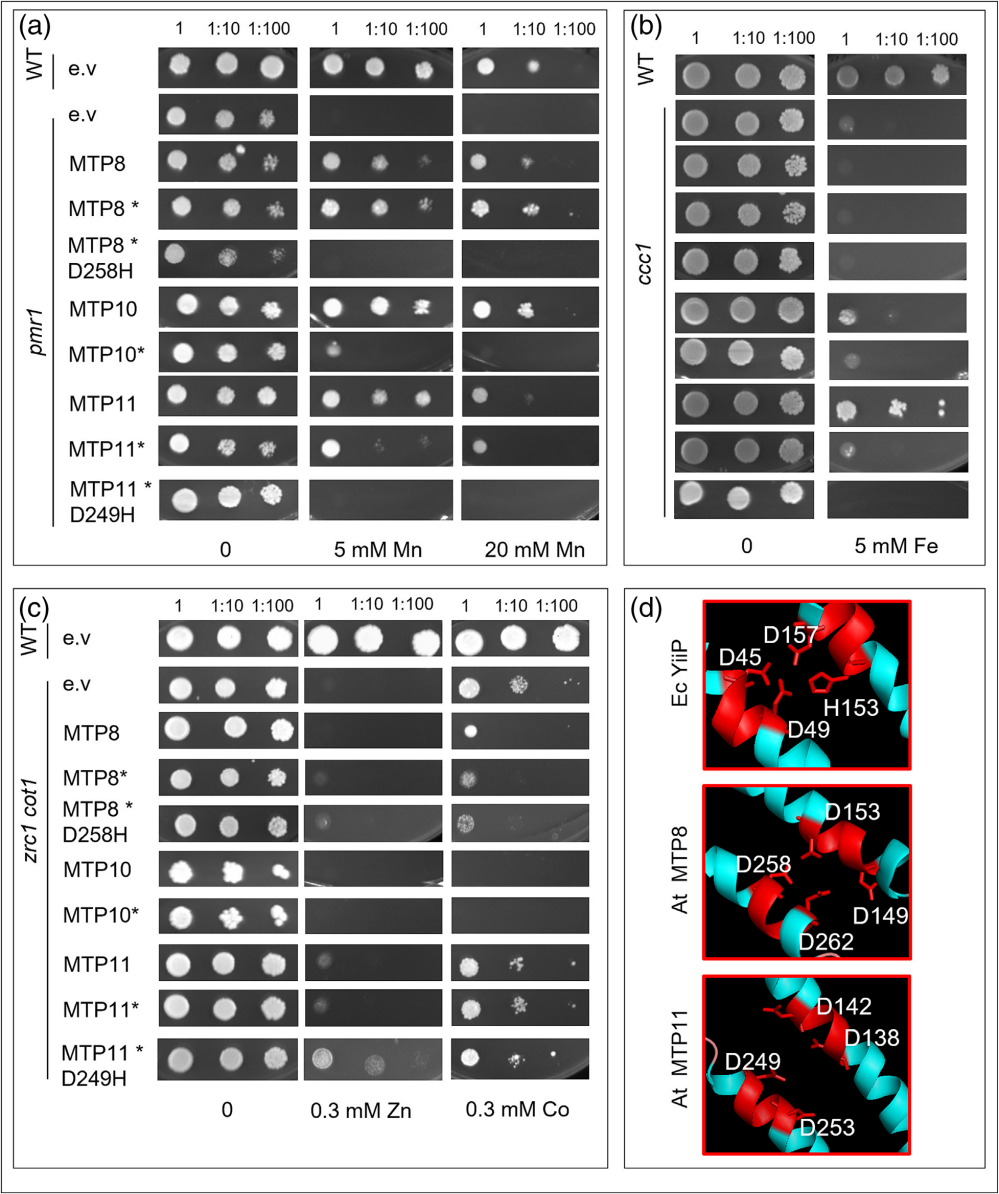
Functional analysis of MTP8, MTP10 and MTP11 in sensitive yeast mutants. (a–b) Expression of MTP8, MTP10 and MTP11 with (*) or without a C‐terminal GFP tag in (a) Mn‐sensitive yeast mutant *pmr1*, (b) Zn‐ and Co‐sensitive *zrc1 cot1*, and (c) Fe‐sensitive *ccc1*. Site‐directed mutants MTP8‐D258H and MTP11‐D249H are also included for comparison. Serial dilutions of yeast cells in liquid SC galactose without uracil: undiluted (1) OD 600 = 0.5, 1:10 and 1:100, dropped onto SC galactose without uracil (control) and supplemented with either (a) MnCl_2_, (b) ZnSO_4_, or CoCl_2_ or (c) FeSO_4_. WT, wild type; e.v., empty pAG426galEGFP vector. Plates were incubated for 5 days at 30°C. (d) Putative Mn^2+^‐binding site A in MTP8 and MTP11, compared with characterized Zn^2+^‐binding site A in EcYiiP. Based on homology model using EcYiiP as template, showing key conserved DxxxD/HxxxD domains on transmembrane domains 2 and 5. Model constructed and visualized using Swiss‐Model (Biasini et al., 2014) and the PyMOL Molecular Graphics System, Version 1.8 Schrödinger, LLC. Full predicted structure in Figure S9.

MTP8, MTP10, and MTP11 target intracellular membranes characteristic of the Golgi when expressed in yeast (Figure S9). Each was expressed in metal‐sensitive yeast mutants to directly compare their ability to restore metal tolerance. Expression of MTP11 was able to restore Mn tolerance in *pmr1* at 5 mM Mn, whereas MTP8 and MTP10 restored WT‐like tolerance up to 20 mM. This supports previous claims of Mn‐transporting ability for MTP8 and MTP11, which were formerly only tested up to 6 and 3 mM Mn, respectively (Eroglu et al., 2016; Peiter et al., 2007), and demonstrates MTP10 is equally effective in rescuing *pmr1*. Tagging MTP10 C‐terminally with GFP interferes with its ability to rescue *pmr1* above 5 mM Mn (Figure 9a). In order to determine MTP8/10/11 specificity for Mn transport, each MTP was also expressed in Zn‐, cobalt‐ (Co) and Fe‐sensitive yeast mutants. None of the MTPs appear able to effectively restore tolerance to Zn‐ and Co‐sensitive *zrc1cot1* (Figure 9c), whereas only non‐tagged MTP11 is able to partially restore tolerance of *ccc1* to Fe (Figure 9b). Previously, MTP8 has been shown to rescue *ccc1* (Chu et al., 2017), which is in contrast to what we observed here.

To explore the importance of the conserved DxxxD domain of TMD 5 in MTP function, site‐directed mutants were generated to substitute the DxxxD of MTP8 and MTP11 for HxxxD, as found in Zn‐transporting MTP1 (MTP8‐D258H and MTP11‐D249H). Expression in yeast indicates that this substitution abolishes Mn transport in both MTP8 and MTP11 compared with the non‐mutated form. D249H in MTP11 appears to confer a very slight resistance to Zn compared with non‐mutated MTP11 in *zrc1cot1*; there was no effect of these mutations on Co or Fe transport (Figure 9a–c). A hypothetical tertiary structure of MTP8 and MTP11 was generated using EcYiiP, the only CDF crystallized to date, as a homology model template (Figure 9d). The DxxxD/HxxxD domains of EcYiiP TMDs 2 and 5, respectively, are reported to form a Zn‐binding site coordinated by the three aspartate and single histidine ions (Lu & Fu, 2007; Lu et al., 2009). Based on this model, the DxxxD domains of MTP8 and MTP11 are also predicted to form a potential ion‐binding pore within the membrane‐bound component of the protein (Figure 9d). The full hypothetical tertiary structures are shown in Figure S9M–O.

## 3 DISCUSSION

### 3.1 The major function of ECA3 is in Mn deficiency

**FIGURE 10:**
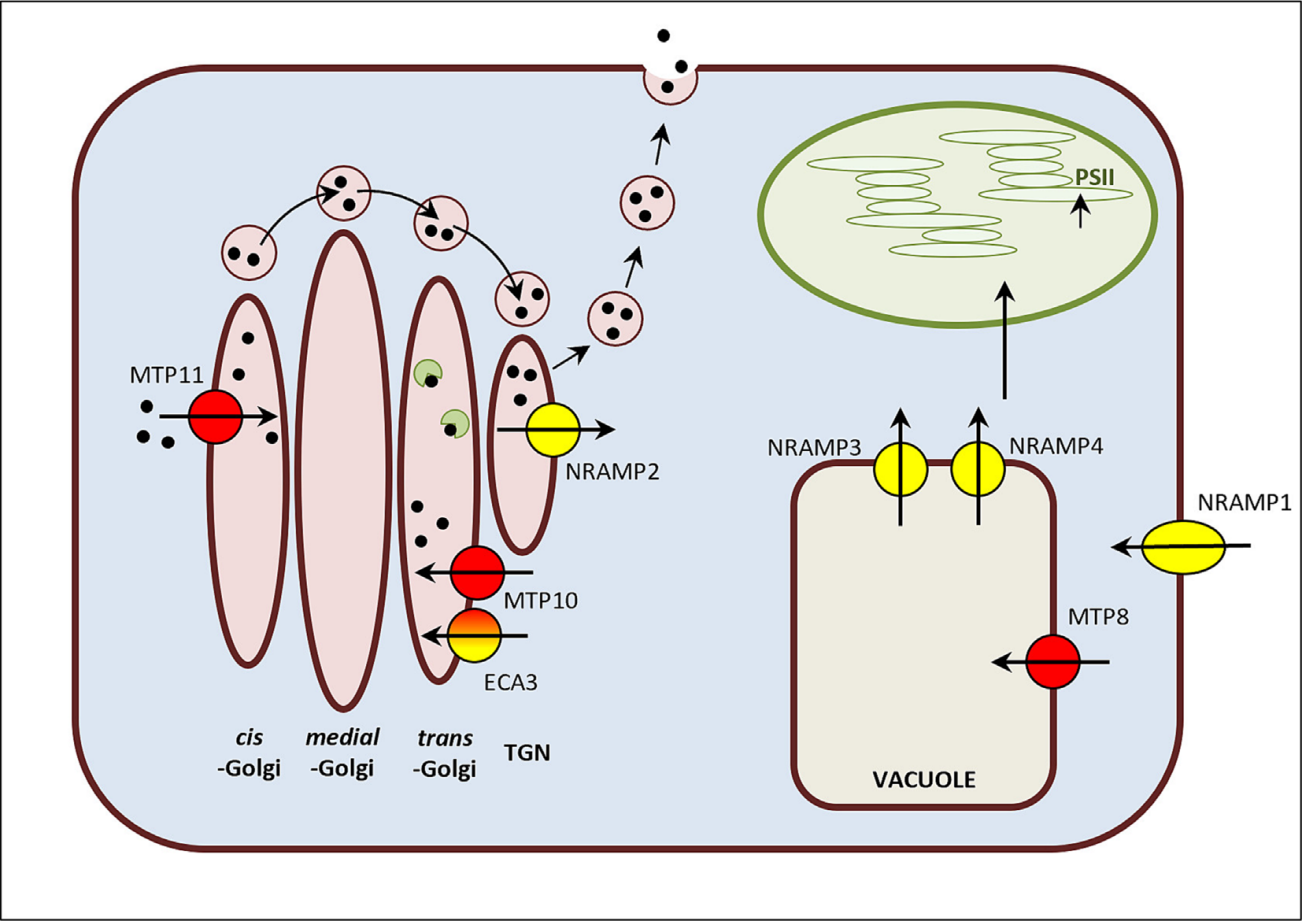
Schematic for subcellular localization of transporters characterized in this study demonstrating their role in Mn homeostasis. Subcellular localization of Arabidopsis transporters determined in this study, including ECA3, MTP11, MTP8, and MTP10. Mn transporters localized previously include, NRAMP1 (Cailliatte et al., 2010), NRAMP2 (Alejandro et al., 2017), NRAMP3, and NRAMP4 (Lanquar et al., 2010). Previous models have suggested that under Mn‐deficiency conditions, NRAMP1 is responsible for Mn import at the plasma membrane and NRAMP2 exports Mn from the TGN, where it is then used in other subcellular compartments, including the vacuole and chloroplasts via NRAMP3 and NRAMP4 (Alejandro et al., 2017). NRAMP2, NRAMP3, and NRAMP4 act together in the process to provide Mn to photosystem II (PSII) (Alejandro et al., 2017). We show that ECA3 is important under Mn deficiency and propose it sequesters Mn into the *trans*‐Golgi for incorporation into and proper glycosylation of important enzymes and proteins (shown in green). Under Mn‐toxic conditions, we use our protein localization data to hypothesis a further model. MTP8 is involved in sequestering toxic Mn from the cytoplasm into the vacuole. Meanwhile, MTP10 and MTP11, which sequester Mn into the *cis*‐ and *trans*‐Golgi, respectively, initiate vesicular trafficking to the plasma membrane for efflux from the cell, as proposed by Peiter et al. (2007). ECA3 also has a minor role (only seen when MTP11 is non‐functional) in alleviating Mn toxicity, where it may sequester toxic Mn from the cytoplasm. Red, involved in sequestration to alleviate Mn toxicity; yellow, involved in sequestration or uptake to alleviate Mn deficiency. ECA3 is proposed to be involved predominantly in Mn deficiency with a minor role under toxicity. Arrow shows proposed direction of Mn transport with respect to the membrane.

A major aim of this investigation was to further understand the ways in which ECA3 and MTP11 transporters contribute to Mn transport and homeostasis in *Arabidopsis*. MTP11 has been shown to be involved in toxicity tolerance (Delhaize et al., 2007; Peiter et al., 2007), but the role of ECA3 is more controversial, with claims that it has a role in either Mn deficiency (Mills et al., 2008) or toxicity (Li et al., 2008). This was addressed here by directly comparing the growth of single mutants at different levels of Mn supply and also generating a double mutant in which both genes were non‐functional. The *eca3‐1*, *eca3‐2*, and *eca3‐4* mutants displayed clear growth defects under Mn deficiency, both under low and standard Ca, thus confirming an important role for ECA3 during low Mn conditions (Mills et al., 2008). Knocking out *mtp11* as well under these conditions did not add to this detrimental effect; if anything, a slight improvement was observed, which could indicate MTP11 may be sequestering the trace amount of Mn that is available for important processes elsewhere. Thus, ECA3 plays an important role in contributing positively to Mn efficiency. A schematic for the roles of ECA3 and MTP11, as well as the other transporters investigated in this study, is shown in Figure 10.

NRAMP1 and NRAMP2 have previously been implicated in Mn transport under deficiency conditions (Alejandro et al., 2017; Cailliatte et al., 2010; Gao et al., 2018), and the stunted growth of *nramp1‐1* and *nramp2‐5* confirms this. The *eca3‐1* mutant showed a similar response to *nramp2‐5* but was more stunted than *nramp1‐1* under Mn deficiency. Additionally, the corresponding double mutants are each additive under Mn deficiency, indicating distinct functions of these transporters. Moreover, the triple *nramp1‐1 nramp2‐5 eca3‐1* triple mutant showed further additive sensitivity in comparison to the *nramp1‐1 nramp2‐5* double mutant. The additive sensitivities correspond with the findings that each protein targets a different subcellular membrane, with NRAMP1 targeting the plasma membrane (Cailliatte et al., 2010), NRAMP2 targeting the TGN (Alejandro et al., 2017; Gao et al., 2018), and ECA3 targeting the *trans‐*Golgi (Figure 10).

### 3.2 ECA3 has a minor role in alleviating Mn toxicity, but only when MTP11 is non‐functional

Li et al. (2008) claimed a role for ECA3 in Mn detoxification, reporting strong inhibition of root growth in *eca3‐4* mutants (50%–60% inhibition compared with WT at 50 μM Mn). We compared all three *eca3* mutants together with WT but did not observe any significant inhibition compared with WT under 50 μM Mn, nor at higher concentrations. *mtp11‐1* is highly sensitive to elevated Mn, and so it is clear that MTP11 is more important than ECA3 under Mn toxicity. However, greater levels of stunting and chlorosis are observed in the *eca3 mtp11* double mutants compared with *mtp11‐1*, suggesting ECA3 does play a minor role in alleviating Mn toxicity, which is only apparent when MTP11 is non‐functional. These findings may suggest ECA3 and MTP11 participate in Mn homeostasis at different pathways. Here, we transiently co‐expressed MTP11 and ECA3 in tobacco, together and with organelle markers, demonstrating that ECA3 targets the *trans‐*Golgi, whereas MTP11 targets the *cis‐*Golgi (Figure 10). This finding favors reports by Peiter et al. (2007), who hypothesized that MTP11 aids Golgi‐based Mn accumulation, leading to vesicular trafficking and exocytosis as a route for Mn detoxification. The Golgi localization of ECA3 also favors findings by Mills et al. (2008). This could imply that the primary role of ECA3 in Mn homeostasis is to alleviate deficiency by supplying Mn to key enzymes in the Golgi. However, this Golgi compartmentalization may also be beneficial under elevated Mn, sequestering potentially toxic Mn from the cytoplasm. This minor role is only apparent when MTP11 is non‐functional, perhaps due to different thresholds of the *trans‐* and *cis‐*Golgi compartments for Mn accumulation. This study also highlights the importance of the Golgi in both extremes of Mn nutrition in plants.

### 3.3 Susceptibility to Mn depends on external Ca concentration

Here, we have shown that the level of Ca supplied also has an effect on the concentration at which Mn toxicity symptoms are observed. When exposed to 100 μM Mn in combination with standard Ca levels (1.495 mM Ca), growth was not detrimentally affected; reducing the Ca concentration caused this Mn concentration to become increasingly toxic to all genotypes but particularly to *mtp11‐1* and *eca3 mtp11*. The greater sensitivity of the *eca3 mtp11* mutant compared with *mtp11‐1* was clearly seen. Similarly, we also observed a Mn‐dependent reduction in germination in *mtp8 mtp11* and *mtp8 mtp10 mtp11* that was apparent under the low Ca conditions.

Addition of Ca can reduce Mn uptake and toxicity in young tomato plants (Gunes et al., 1998), peanut plants (Bekker et al., 1994), and barley (Alam et al., 2006). Elevated Ca can also lead to greater Fe accumulation in barley by alleviating Mn‐induced Fe deficiency (Alam et al., 2006). However, there is little understanding of the mechanisms involved in this Ca/Mn antagonism. Other findings could suggest there is an additional entry pathway for Ca, for which Mn can compete, or vice versa. Although some Ca channels are highly selective, nonselective cation channels also exist, allowing divalent cations to permeate into the cytoplasm (for review, see Demidchik & Maathiuis, 2007); however, the ability to transport Mn by Ca channels is not always tested. It is possible that under low Ca, Mn could compete for less specific Ca pathways to enter the root cells and cause cytoplasmic toxicity. Alternatively, Ca could compete with Mn for subcellular targets, a competition that, under basal conditions, would alleviate the toxic effect of Mn. There is little information available regarding Mn/Ca displacement, but there are examples of other elements competing with Ca for active sites in proteins. For example, lanthanides can compete with Ca in the water splitting/oxygen evolving complex of PSII, Mn_4_CaO_5_ (Ghanotakis et al., 1985). Ca can be replaced with other metals in this protein and retain the core structure, but Ca is essential for proper function due to its role in organizing the water network surrounding the protein (Lohmiller et al., 2012).

### 3.4 MTP8, MTP10, and MTP11 all contribute to Mn tolerance, as concluded from a Mn‐hypersensitive triple mutant

The results from our comparative study of *mtp* mutants indicate that MTP8, MTP10, and MTP11 all contribute to alleviating Mn toxicity. On standard and low‐Ca media, knocking out *MTP11* has the most pronounced detrimental effect on growth and chlorophyll levels, with a milder inhibition seen in the *mtp8* mutant. The effect of knocking out *MTP10* is only significantly noticeable in the triple *mtp8 mtp10 mtp11* mutant when both MTP11 and MTP8 are also non‐functional, highlighting the importance of generating double and triple mutants to investigate transporter function. An underlying role of MTP10 in alleviating Mn toxicity is further observed when overexpressed in *mtp11‐1,* where MTP10 was able to partially restore the Mn‐sensitive growth of the mutant. The mitigating role of MTP10 in this scenario indicates that under high expression levels, MTP10 is able to perform a similar role to MTP11. The lack of full rescue, however, suggests that the function of these different MTP proteins is discrete within the plant. When expressed stably in *Arabidopsis* and transiently in tobacco, MTP10 targets the *trans‐*Golgi, whereas MTP11 targets the *cis‐*Golgi, again providing evidence for the importance of the Golgi in Mn homeostasis (Figure 10).

MTP8 is confirmed as important in alleviating Mn toxicity under conditions of low Fe and elevated pH (Eroglu et al., 2016). Importantly, we show that MTP8 is the only Mn‐MTP involved in providing this tolerance; the other single *mtp* mutants remain unaffected and knocking out MTP10 and MTP11 in addition to MTP8 has no additional marked effect.

### 3.5 Overexpression of *MTP8* in *Arabidopsis* conferred enhanced tolerance to Mn toxicity

When we stably expressed MTP8‐GFP in *Arabidopsis*, and transiently in tobacco cells, fluorescence is characteristic of the tonoplast. The MTP8 signal runs continuously, internally to chloroplast autofluorescence, and forms transvacuolar strands to enable passage of the Golgi and cytoplasmic contents across the cell. A tonoplast localization has previously only been reported when MTP8 is transiently expressed in mesophyll protoplasts and tobacco leaves (Eroglu et al., 2016; Zhang et al., 2021), but it was important to confirm this in intact *Arabidopsis* cells as localization for MTP8 homologs has been shown to vary. *Stylosanthes hamata* MTP1 and *Oryza sativa* MTP8 have been shown to localize to the tonoplast (Chen et al., 2013; Delhaize et al., 2003), but *H. vulgare* MTP8.1 and HvMTP8.2 are reported to target the Golgi (Pedas et al., 2014), and *Camellia sinensis* MTP8 (tea) has been shown to target the plasma membrane (Li et al., 2017).

We also confirm that overexpression of *MTP8* in *Arabidopsis* confers enhanced tolerance to elevated Mn levels (higher biomass and chlorophyll per seedling) under basal Ca and also show this in low Ca conditions. MTP8‐overexpressing plants have previously shown increased tolerance to moderate Mn toxicity (Chu et al., 2017) and enhanced Mn accumulation in root vacuoles (Eroglu et al., 2016). Taken together, these findings support the hypothesis that the hypertolerance to Mn toxicity conferred by MTP8 overexpression is due to enhanced Mn sequestration within the vacuole, enabling greater resistance to cytoplasmic toxicity and thus enhancing growth (Figure 10). This mechanism is also in agreement with results for *Glycine max* (soybean) MTP8, which also confers resistance to elevated Mn when expressed in *Arabidopsis* (Li et al., 2021). In this case, GmMTP8 was localized to the ER, although localization was not shown in plants that were conferring Mn tolerance (Li et al., 2021). In contrast, although tea CsMTP8 confers increased tolerance in *Arabidopsis*, it is proposed to function in Mn transport out of the cell as it is localized to the plasma membrane and reduces Mn accumulation (Li et al., 2017). Nevertheless, whichever mechanism, the results suggest that developing crops with enhanced expression of *MTP8* could be beneficial in enabling survival and improving yield when grown under poor soil conditions.

### 3.6 Heterologous expression in yeast supports a Mn‐transporting role for Arabidopsis Group 8/9 CDFs

MTP10 and MTP11 both showed a punctate, Golgi‐like pattern in yeast consistent with the localization seen in plants. Unlike the tonoplast localization *in planta*, we found that MTP8 localized to a punctate endomembrane compartment in yeast. A similar mislocalization has been observed for ShMTP8, which also targets the tonoplast in *Arabidopsis* and tobacco, but localizes in this case to the ER in yeast (Delhaize et al., 2003). It is possible, therefore, that the sorting signals for some of the Mn‐MTPs may not be recognized appropriately in yeast, although the system is still extremely useful in testing for Mn transport properties. When compared directly, MTP8 and MTP10 conferred the greatest Mn tolerance to yeast mutant *pmr1*, whereas MTP11, although still providing tolerance, was not quite as effective. MTP8 and MTP10 seem specific for Mn, whereas MTP11 also conferred slight Fe tolerance to *ccc1*, suggesting an ability to also transport Fe. In contrast, Chu et al. (2017) reported that MTP8, but not MTP11, was able to restore growth of *ccc1*. Here Fe was supplied at 5 mM, whereas Chu et al. (2017) supplied 3 mM Fe, so this may account for this difference.

Most hypothetical structural information for CDFs is based on the crystallized structure of Zn‐transporting CDF, EcYiiP. EcYiiP functions as a homodimer with three Zn‐binding sites, A, B, and C. Zn ions at site A are coordinated by the DxxxD and HxxxD motifs of TMDs 2 and 5, respectively, which form the DD‐HD coordination site (Lu & Fu, 2007; Lu et al., 2009). These domains are conserved between all CDFs, suggesting this site is also conserved, although the exact residues vary between kingdoms (Montanini et al., 2007). Based on an EcYiiP homology model, the DxxxD domains on TMDs 2 and 5 of MTP8 and MTP11 are predicted to form a DD‐DD ion‐coordination site (Figure 9d). These motifs are substituted for HxxxD domains in most plant Zn‐transporting MTPs (Montanini et al., 2007). As such, MTP8‐D258H and MTP11‐D249H were generated, substituting the TMD5 DxxxD for HxxxD and the putative DD‐DD site for DD‐HD. This mutation abolishes the ability of MTP8 and MTP11 to transport Mn, with a very slightly improved Zn‐tolerance for MTP11 in *zrc1cot1* (Figure 9a–c). These mutations reveal significant information about the importance of this domain in Mn‐MTP function, although it shows that more residues are likely involved in maintaining specificity and that further modifications are required to completely alter the affinity of Mn‐MTPs. Site‐directed mutations that lead to a loss of function have recently indicated the importance of these domains in Mn‐transporting OsMTP8.1 (Chen et al., 2016), Os MTP11 (Farthing et al., 2017), and the human SLC30A10 (Nishito et al., 2016; Zogzas et al., 2016). However, here we go further in substituting a residue conserved in Mn‐MTPs for that conserved in Zn‐MTPs, to investigate its importance in maintaining substrate specificity. Increased understanding of the mechanisms that maintain this specificity may have agricultural implications in the future.

## 4 CONCLUSION

This study concludes that ECA3 plays a major role in alleviating Mn deficiency, with a minor role in Mn toxicity. Direct comparisons of ECA3 alongside NRAMP1 and NRAMP2 mutants indicate that all three have important and distinct roles in responding to Mn deficiency. The study highlights the importance of comparing mutants, and generating double and triple mutants, to assess the relative contribution of different transporters in Mn homeostasis. Although the importance of MTP8 and MTP11 is revealed in the single knockout mutants, the contributions of ECA3 and MTP10 to Mn toxicity are only evident in double and triple mutants and overexpressor lines. An additive phenotype is observed in *eca3 mtp11*, whereas MTP10 shows its contribution to the Mn toxicity response via the severe Mn‐dependent phenotype of *mtp8 mtp10 mtp11* as well as the rescue of the Mn‐sensitive *mtp11* mutant. This study also demonstrates the importance of intracellular compartmentalization in protecting *Arabidopsis* against Mn toxicity, with Group 8/9 CDFs playing important roles. Yeast metal‐tolerance assays support a role for MTP10 as a Mn transporter, which has not been reported previously, and indeed, MTP8 and MTP10 are both more effective in restoring Mn tolerance in yeast at higher levels than MTP11. MTP10 and MTP11 appear to localize to different regions of the Golgi in plants, whereas MTP8 is tonoplast‐localized, potentially providing three different routes for Mn detoxification in the plant cell. Overexpression of MTP8, shown here to confer hypertolerance to Mn toxicity conditions, indicates that these proteins could bestow useful properties if expressed in crops, allowing plants to resist unfavorable nutrient conditions.

## 5 METHODS

### 5.1 Growth of *Arabidopsis* plants

*Arabidopsis thaliana* plants were in the Columbia (Col) WT background. Soil‐grown plants were grown as described in Menguer et al. (2013). To achieve soil of pH 7.2, soil was limed with 20 g/kg CaCO_3_ and 12 g/kg NaHCO_3_; pH was determined using 100 mM CaCl_2_. Non‐limed soil was pH 6.0.

### 5.2 Isolation of T‐DNA insertion mutants

Single T‐DNA mutants have previously been described for *eca3‐1* (N545567), *eca3‐2* (N570619; Mills et al., 2008), *eca3‐4* (SALK_032802; Li et al., 2008), *mtp11‐1* (SALK_525517), *mtp8‐1* (SALK_068494), *mtp8‐2* (SALK_140266; Eroglu et al., 2016), and *nramp1*‐*1* (Cailliatte et al., 2010). In addition to these, insertion mutant lines for *mtp10‐1* (SALK_121470), *mtp10‐2* (SALK_023321), and *nramp2‐5* (WiscDsLoxHs005_04F) were obtained from the Nottingham Arabidopsis Seed Centre (NASC; www.arabidopsis.info) and homozygous mutants isolated for all. Single mutants were crossed to obtain the following double mutants: *eca3‐1 mtp11‐1*, *eca3‐2 mtp11‐1*, *mtp8‐2 mtp11‐1*, *mtp8‐2 mtp10‐1*, *mtp10‐1 mtp11‐1*, *eca3‐1 nramp1‐1*, *eca3‐1 nramp2‐5*, *and nramp1‐1 nramp2‐5*. The triple homozygous mutant *mtp8‐2 mtp10‐1 mtp11‐1* was obtained after crossing *mtp8‐2 mtp10‐1* and *mtp8‐2 mtp11‐1*. The triple homozygous mutant *nramp1‐1 nramp2‐5 eca3‐1* was obtained after crossing *eca3‐1 nramp2‐5* and *nramp1‐1 nramp2‐5*. To confirm homozygous mutants, genomic DNA was isolated using the DNAMITE plant kit (Microzone Ltd.; www.microzone.co.uk), and RNA was extracted using a phenol/LiCl precipitation method (based on Verwoerd et al., 1989) or a Trizol‐based method (Life Technologies Ltd; www.thermofisherscientific.com). One microgram RNA was used for first‐strand cDNA synthesis using ImPromp‐II Reverse Transcriptase (Promega; www.promega.co.uk). PCR with primers that span the insert site were used to confirm lack of product or transcript at genomic and RNA level, respectively, and primers that target the gene and the T‐DNA to confirm presence of T‐DNA at the genomic level (primers to confirm lack of expression are listed in Table S1).

### 5.3 Generating DNA constructs for MTP8, MTP9, MTP10, and MTP11

The cDNA sequences for *Arabidopsis MTP8* (At3g58060), *MTP9* (At1g79520), *MTP10* (At1g16310), and *MTP11* (At2g39450) are listed on TAIR (Lamesch et al., 2011). *MTP9*, *MTP10*, and *MTP11* sequences were amplified from WT Columbia cDNA with and without the stop codon, using Pfu polymerase (Promega), adding an N‐terminal CACC tag for cloning into the pENTR/D‐TOPO vector (Invitrogen; www.thermofisherscientific.com) (“TOPO” primers listed in Table S1). To obtain *MTP8*, full sequence lacking the first 11 bases was amplified using MTP8F2 and MTP8_toponostop; this was extracted using QIAquick gel extraction kit (Qiagen; www.qiagen.com) and used as template to amplify full‐length *MTP8* with primers MTP8_topoF2 and MTP8_toponostop. Site‐directed mutations for MTP8‐D258H and MTP11‐D249H were generated using QuikChange II XL Site‐Directed Mutagenesis Kit (Agilent Technologies; www.genomics.agilent.com). Mutational primers were RP1‐purified (Sigma Aldrich) and are listed in Table S1. All other primers were obtained from IDT‐DNA (eu.idtdna.com).

Sequences of entry clones in pENTR/D‐TOPO were confirmed and *MTP8*, *MTP10*, and *MTP11* sequences were recombined with destination vectors (using Gateway LR Clonase II™ enzyme; Invitrogen). For example, plant‐expression constructs Pro35S::MTP8 and Pro35S::MTP8::GFP were generated using pMDC32 and pMDC83 vectors (Curtis & Grossniklaus, 2003); Pro35S::MTP11::mRFP was generated using pSITE‐4NB vector (Chakrabarty et al., 2007); Pro35S::YFP::ECA3 was generated using pEG104 (Earley et al., 2006) as previously described (Mills et al., 2008). For yeast expression, pAG426GAL‐ccdB‐EGFP (Alberti et al., 2007) was used to generate ProGAL::MTP8::GFP, ProGAL::MTP10::GFP and ProGAL::MTP11::GFP. All destination clones were confirmed with sequencing.

### 5.4 Plate‐based metal tolerance assay in *Arabidopsis*

*Arabidopsis* seeds were surface‐sterilized in 15% (v/v) bleach for 20 min, rinsed five times with sterile water, and inoculated onto plates containing 0.8% (w/v) agarose (Melford Laboratories Ltd; www.melford.co.uk), 1% (w/v) sucrose (VWR Chemicals; uk.vwr.com), and either one‐half‐strength Murashige and Skoog medium (1/2 MS; Murashige & Skoog, 1962) or the same medium but with Ca levels ranging from 1.495 mM (standard Ca) to 0.1 mM CaCl_2_ (low Ca) as described previously (Mills et al., 2008). For Mn assays, Mn was supplied as MnSO_4_ at the indicated concentrations, and for Mn deficiency, no Mn salts were added to the media. Fe/pH experiments were set up as described in Eroglu et al. (2016). Seed were stratified at 4°C for 48 h prior to transfer to a controlled‐environment cabinet (23°C, 16 h light; 18°C, 8 h dark; light intensity 100–120 μmoles m^−2^ s^−1^) with plates incubated vertically. Plants were grown for 21–24 days. Fresh weight (FW) and chlorophyll measurements were determined as described previously (Menguer et al., 2013; Mills et al., 2008), with generally six plates per condition, with four seedlings per genotype, per plate. Chlorophyll was determined following extraction in *N*,*N*‐dimethylformamide (Moran, 1982; Sigma‐Aldrich; www.sigmaaldrich.com). Experiments presented are representative of at least two independent experiments, and data are the means ± SE, on a per‐seedling basis. All measurements were analyzed using one‐way ANOVA or GLM at each concentration, with Tukey's post hoc test.

### 5.5 *Arabidopsis* transformation

GV3101 *Agrobacterium tumefaciens* cells carrying plant expression vectors were used to transform *Arabidopsis* WT (Columbia), *mtp8‐2*, and *mtp11‐1* plants using the floral dip method (Clough & Bent, 1998). Positive transformants were selected on ½ MS plates supplemented with 50 μg mL^−1^ hygromycin. Segregation analyses at the T2 and T3 stages were performed to isolate single‐insertional homozygous transgenic plants. Metal tolerance assays and confocal microscopy studies were performed on homozygous T3 plants. To image, whole seedlings were placed on a microscope slide in water with a cover slip. Cell wall staining was performed with propidium iodide (Invitrogen), described in Menguer et al. (2013). Representative images are presented, after imaging several independent transgenic lines, with multiple seedlings imaged per line.

### 5.6 Real‐time PCR

*Arabidopsis* material was harvested for RNA extraction and cDNA synthesis as above. Quantitative real‐time PCR (qPCR) was carried out in an Applied Biosystems StepOne real‐time cycler using PrecisionPLUS Rox and SYBR Green Mastermix (Primer Design). Relative expression levels of *MTP8* and *MTP10* were determined according to Pfaffl (2001) and normalized against *UBQ10* as a constitutively expressed control. Primers are listed in Table S1.

### 5.7 Transient expression in tobacco

GV3580 *A. tumefaciens* cells carrying fluorescent‐tagged plant expression vectors were inoculated overnight in selective LB. Cultures were washed twice and resuspended to a final OD_600_ of 0.1–0.5 in infiltration medium (50 mM MES, pH 5.6; .5% (w/v) D‐Glucose; 2 mM Na_3_PO_4_; 60 mg/L acetosyringone in dimethyl sulfoxide). This *Agrobacterium* suspension was infiltrated into 4‐ to 6‐week‐old greenhouse‐grown tobacco plants (*Nicotiana tabacum*, Petit Havana), either WT or stably transformed with *trans‐*Golgi‐marker ST::RFP, as described in Brandizzi et al. (2002). ManI::GFP in the pBI221 vector (Shen et al., 2013) was used as a *cis‐*Golgi co‐expression marker. Infiltrated plants were grown normally for a further 48 h; 1‐cm leaf‐tissue discs were mounted in water on a microscope slide with cover slide and fluorescence observed using confocal microscopy as below. Representative images are presented, after at least three independent transfections per construct.

### 5.8 Yeast transformation and metal‐tolerance assays

*Saccharomyces cerevisiae* yeast strains used were: WT BY4741 and *pmr1* (Euroscarf; www.euroscarf.de) for Mn‐complementation analyses or *zrc1 cot1* for Zn‐ and Co‐complementation analyses; WT DY150 and *ccc1* were used for Fe‐complementation analyses. Full genotype information is listed in Menguer et al. (2013). Yeast transformation was performed using a LiOAc/PEG method (Gietz et al., 1992) selected on SC (synthetic complete) medium without uracil and with 2% (w/v) glucose, as described in Menguer et al. (2013).

Yeast cultures were inoculated overnight at 30°C in 5 mL SC glucose without uracil then to induce expression of genes of interest; cultures were resuspended in SC media without uracil, with 2% (w/v) galactose in place of glucose, and incubated for a further 4 h before dilution to OD_600_ = 0.4. Further serial dilutions of 1/10 and 1/100 were also made. Seven μL culture was dropped onto 2% agar (w/v) plates containing SC galactose medium without uracil, supplemented with a range of metal concentrations (MnCl_2_, ZnSO_4_, CoCl_2_, FeSO_4_; all Sigma‐Aldrich). Plates were incubated at 30°C for 5 days.

### 5.9 Localization studies in yeast

In‐frame C‐terminal fusions of ProGAL::MTP8::GFP, ProGAL::MTP10::GFP, and ProGAL::MTP11::GFP were transformed in BY4741 WT. Site‐directed MTP8 and MTP11 mutants were also transformed. To induce expression, cultures were grown as above. Cultures were fixed by resuspending in 100 μL 4% paraformaldehyde at room temperature for 15 min, before washing twice and resuspending in 100 μL of a 1 M KH_2_PO_4_ /1 M K_2_HPO_4_/2 M sorbitol mix. Three microliter cells were positioned on a microscope slide with cover slip and imaged as below.

### 5.10 Confocal fluorescent imaging

Fluorescence was observed using a Leica SP8 confocal laser scanning microscope. GFP excitation, 488 nm; detection, 500–540 nm. RFP and propidium iodide excitation, 561 nm; detection, 565–600 nm. Chlorophyll autofluorescence excitation, 633 nm; detection, 650–700 nm.

### 5.11 Phylogenetic and sequence analysis

Multiple sequence alignments were performed by Clustal Omega (Sievers et al., 2011). Transmembrane domains were predicted using AramTmConsens, a consensus transmembrane alpha helix prediction program that combines output from 18 individual prediction programs, available on the ARAMEMNON database (Schwacke et al., 2003). For phylogenetic analysis, sequences for protein homologs to Arabidopsis MTP8–MTP11 were obtained from: *Populus trichocarpa* (poplar) and *Sorghum bicolor* (sorghum), Gustin et al. (2011); *Beta vulgaris* spp. *maritima*, Erbasol et al. (2013); *O. sativa* (rice), Chen et al. (2013); HvMTP8 and HvMTP8.1 from *H. vulgare* (barley), Pedas et al. (2014). *Arabidopsis* and rice sequences were used to search for homologs in *B. rapa*, *Brachypodium distachyon*, and *Zea mays* on Phytozome v9.1 (Goodstein et al., 2012), *Cucumis sativus* and *Vitis vinifera* on EnsemblPlants (Howe et al., 2020) and in barley on the International Barley Sequencing Consortium database (Schulte et al., 2009). The HvMTP11 coding sequence was predicted from the HvAK372762.1 contiguous sequence. A phylogenetic tree was reconstructed with Neighbor‐Joining method, performed using MEGA (Molecular Evolutionary Genetics Analysis) 7 package (Kumar et al., 2016) with the following parameters: 1000 bootstrap replicates, pairwise deletion, and Poisson correction. Sequence data from this article can be found in data libraries under accession numbers listed in Table S2.

Hypothetical structural models for MTP8 and MTP11 were constructed using Swiss‐Model (Biasini et al., 2014), with EcYiiP as homology template. Structures were visualized using The PyMOL Molecular Graphics System, Version 1.8 Schrödinger, LLC.

## AUTHOR CONTRIBUTIONS

Lorraine E. Williams conceived the study. Emily C. Farthing, Kate C. Henbest, Tania Garcia‐Becerra, and Kerry A. Peaston conducted the experiments. Lorraine E. Williams, Emily C. Farthing, and Kate C. Henbest analyzed the data and wrote the paper.

## CONFLICT OF INTEREST STATEMENT

The Authors did not report any conflict of interest.

## Supporting information


**Figure S1.** **Confirmation of *eca* and *nramp* Arabidopsis mutants at RNA level. Related to Figure 1.** Confirmation of (A) *eca3‐1, nramp1‐1* and *eca3‐1 nramp1‐1*, (B) *nramp2‐5* and *nramp1‐1 nramp2‐5*, (C) *eca3‐1 nramp2‐5* single and double mutants and (D) the *nramp1‐1 nramp2‐5 eca3‐1* triple mutant. Actin (*ACT2*) is amplified as a control from all samples. *ECA3*, *NRAMP1* and *NRAMP2* products are amplified from WT cDNA samples prepared from reverse transcribed RNA but are absent from relevant single, double and triple mutants. Sizes of molecular weight marker on left; predicted sizes of amplified fragments listed on right of gels.
**Figure S2. Confirmation of *eca* and *mtp* Arabidopsis mutants at RNA level. Related to Figure 2.** Confirmation of (A) *eca3‐1 mtp11‐1* and *eca3‐2 mtp11‐1*, (B) *mtp8‐2 mtp10‐1*, (C) *mtp8‐2 mtp11‐1*, (D) *mtp10‐1 mtp11‐1* double mutants and (E) *mtp8‐2 mtp10‐1 mtp11‐1* triple mutants. Actin (*ACT2*) is amplified as a control from all samples. *ECA3*, *MTP8*, *MTP10* and *MTP11* products are amplified from WT cDNA samples prepared from reverse transcribed RNA but are absent from relevant single, double and triple mutants. Sizes of molecular weight marker on left; predicted sizes of amplified fragments listed on right of gels.
**Figure S3. Three *eca3* insertion mutants are sensitive to Mn deficiency, but not to Mn toxicity compared to WT. Related to Figure 2.** Average fresh weight (FW; mg) and average chlorophyll (Chl; μg) per seedling of Col8 WT, *eca3‐1, eca3‐2* and *eca3‐4* mutants when grown for 20 days on ½ MS supplemented with a range of MnSO_4_ concentrations, and either (A) 1495 μM CaCl_2_ (basal Ca) or (B) 100 μM CaCl_2_ (low Ca). Data shows mean FW (mg) per seedling (±SE) calculated for 6 plates, with 4 seedlings per genotype per plate. Statistical significance was assessed with two‐way ANOVA and Tukey *post‐hoc* test. Means not sharing a letter at a particular condition are significantly different. Photographs display representative growth under different Mn conditions. White bar = 1 cm.
**Figure S4. Evolutionary relationship of putative Mn‐MTPs from different plant species shows clustering into 3 main sub‐clades. Related to Figure 4 and Supplemental Table 2.** Red, Group 8 MTP8 and MTP8.1; green, Group 9 MTP11 and MTP11.1; blue, Group 9 MTP9 and MTP10. Arabidopsis MTP1, MTP6 and MTP7 included as controls for other MTP sub groups. Evolutionary relationships inferred using the Neighbour‐Joining method; bootstrap consensus inferred from 1000 replicates and is taken to represent the evolutionary history of the proteins analysed. The evolutionary distances were computed using the Poisson correction method and are in the units of the number of amino acid substitutions per site. The analysis involved 56 amino acid sequences. All ambiguous positions were removed for each sequence pair. Evolutionary analyses were conducted in MEGA7 phylogenetic analysis package (Kumar et al., 2016). Protein sequences obtained from: *Arabidopsis thaliana* (At), *Brassica rapa* (Br), *Beta vulgaris* spp. maritima (Bm), *Populus trichocarpa* (Pt), *Sorghum bicolor* (Sb), *Brachypodium distachyon* (Bd), *Cucumbis sativis* (Cs), *Oryza sativa* (Os), *Zea mays* (Zm), *Hordeum vulgare* (Hv), *Vitis vinifera* (Vv), *Stylosanthes hamata* (Sh).
**Figure S5. Insertion sites for T‐DNA mutants for *MTP8, MTP10* and *MTP11*, confirmed by sequencing, labelled on genomic schematic. Schematic for *MTP9* genomic is included for comparison. Related to Figure 4 and 6.** Black box, exon; white box, intron; orange box, 5′ and 3 ′ untranslated regions, obtained from TAIR. Arrow, insertion site for *mtp* mutant confirmed by sequencing.
**Figure S6. Multiple sequence alignment of MTP8, MTP9, MTP10 and MTP11. Related to Figure 4 and 6.** Adapted from that generated by ClustalOmega (Seivers et al., 2011). (*) fully conserved residues between sequences; (:) conservation of residues with strongly similar properties; (.) conservation of residues with weakly similar properties. Transmembrane domains (TMDs; highlighted in black) predicted by AramTmConsens (Schwacke et al., 2003). CDF signature sequence marked by red letters. DxxxD domains of Mn‐CDFs underlined in red at TMDs 2 and 5.
**Figure S7. Increased susceptibility of *mtp8 mtp11* and *mtp8 mtp10 mtp11* under low Ca conditions. Related to Figure 4 and 6.** Comparison of Col8, WT and *mtp11‐1* with either *mtp8‐2* and *mtp8‐2 mtp11‐1* (A) or *mtp8‐2 mtp11‐1* and *mtp8‐2 mtp10‐1 mtp11‐1* (B) under Mn toxicity, low Ca conditions. Plants were grown for 24 days on ½ MS supplied with 100 μM CaCl_2_ and a range of MnSO_4_ concentrations. The *mtp10‐1 mtp11‐1* (C) and *mtp8‐2 mtp10‐1* (D) double mutants do not show increases susceptibility to Mn toxicity when compared to WT and single mutants, under basal Ca conditions. Plants were grown for 21 days on ½ MS supplied with 1495 μM CaCl_2_ and a range of MnSO_4_ concentrations. Data shows mean fresh weight (FW; mg) calculated for 6 plates (±SE) with 4 seedlings per genotype per plate. Statistical significance was assessed with two‐way ANOVA and Tukey *post‐hoc* test. Means not sharing a letter at a particular condition are significantly different. Photographs display representative growth under different Mn conditions. White bar = 1 cm.
**Figure S8. Germination rate of *mtp8‐2 mtp11‐1* decreases at high Mn concentrations. Related to Figure 4 and 6.** Comparison of Col8, WT, *mtp11‐1* and *mtp8‐2 mtp11‐1* germination rates under Mn toxicity, low Ca conditions. Plants were grown for 24 days on ½ MS supplied with 100 μM CaCl_2_ and a range of MnSO_4_ concentrations. *mtp8‐2 mtp11‐1* mutants show decreased germination rates at high Mn concentrations. Data shows mean fresh weight (FW; mg) calculated for 6 plates (±SE) with 4 seedlings per genotype per plate. Statistical significance was assessed with two‐way ANOVA and Tukey *post‐hoc* test. * = Means are significantly different to WT.
**Figure S9. MTP8, MTP10 and MTP11 target intracellular membranes when expressed in yeast. Hypothetical tertiary structure of MTP8, MTP11 and EcYiiP. Related to Figure 9.**Differential interference contrast (DIC), GFP fluorescence and overlay of DIC and fluorescence of BY4741 yeast transformed with (A‐C) empty pAG426galEGFP vector, (D‐F) PGAL::MTP8::EGFP, (G‐I) PGAL::MTP10::EGFP and (J‐L) PGAL::MTP11::EGFP. White scale bar = 5 μm. (M‐O) Hypothetical tertiary structure of MTP8 and MTP11, compared with EcYiiP. Based on homology model using EcYiiP as template. Model constructed and visualised using Swiss‐Model (Biasini et al., 2014) and The PyMOL Molecular Graphics System, Version 1.8 Schrödinger, LLC.
**Table S1.** Primers used in this study to confirm zygosity of single, double and triple insertion mutants and to amplify coding sequences for cloning.

**Movie S1.** **MTP8 targets the tonoplast when stably expressed in Arabidopsis. Related to Figure 6 and 7.** Timelapse movie of P35S::MTP8::GFP (green signal) expressed stably in Arabidopsis and imaged after 5 days of growth on ½ MS. Movie shows formation of transvacuolar strands (TVS) and movement of TVS across cell. Interval = 1.4 seconds.

**Table S2.** Accession numbers or sequence codes for protein sequences used in phylogenetic analysis, with original reference or database obtained. TAIR, The Arabidopsis Information Resource. IBSC, the International Barley Consortium Database. Related to tree in Supplemental Figure 3.
