# Supplementary material for: Dissecting the relative contribution of ECA3 and group 8/9 cation diffusion facilitators to manganese homeostasis in Arabidopsis thaliana
Source: Plant Direct. 2023 May 22;7(5):e495. doi: 10.1002/pld3.495 (PMC10202827; doi:10.1002/pld3.495)
Supplement: Supplementary file 1 — Figure S1. Confirmation of eca and nramp Arabidopsis mutants at RNA level. Related to Figure 1. Confirmation of (A) eca3‐1, nramp1‐1 and eca3‐1 nramp1‐1, (B) nramp2‐5 and nramp1‐1 nramp2‐5, (C) eca3‐1 nramp2‐5 single and double mutants and (D) the nramp1‐1 nramp2‐5 eca3‐1 triple mutant. Actin (ACT2) is amplified as a control from all samples. ECA3, NRAMP1 and NRAMP2 products are amplified from WT cDNA samples prepared from reverse transcribed RNA but are absent from relevant single, double and triple mutants. Sizes of molecular weight marker on left; predicted sizes of amplified fragments listed on right of gels. Figure S2. Confirmation of eca and mtp Arabidopsis mutants at RNA level. Related to Figure 2. Confirmation of (A) eca3‐1 mtp11‐1 and eca3‐2 mtp11‐1, (B) mtp8‐2 mtp10‐1, (C) mtp8‐2 mtp11‐1, (D) mtp10‐1 mtp11‐1 double mutants and (E) mtp8‐2 mtp10‐1 mtp11‐1 triple mutants. Actin (ACT2) is amplified as a control from all samples. ECA3, MTP8, MTP10 and MTP11 products are amplified from WT cDNA samples prepared from reverse transcribed RNA but are absent from relevant single, double and triple mutants. Sizes of molecular weight marker on left; predicted sizes of amplified fragments listed on right of gels. Figure S3. Three eca3 insertion mutants are sensitive to Mn deficiency, but not to Mn toxicity compared to WT. Related to Figure 2. Average fresh weight (FW; mg) and average chlorophyll (Chl; μg) per seedling of Col8 WT, eca3‐1, eca3‐2 and eca3‐4 mutants when grown for 20 days on ½ MS supplemented with a range of MnSO4 concentrations, and either (A) 1495 μM CaCl2 (basal Ca) or (B) 100 μM CaCl2 (low Ca). Data shows mean FW (mg) per seedling (±SE) calculated for 6 plates, with 4 seedlings per genotype per plate. Statistical significance was assessed with two‐way ANOVA and Tukey post‐hoc test. Means not sharing a letter at a particular condition are significantly different. Photographs display representative growth under different Mn conditions. White bar = 1 cm. [file PLD3-7-e495-s002.docx]

**
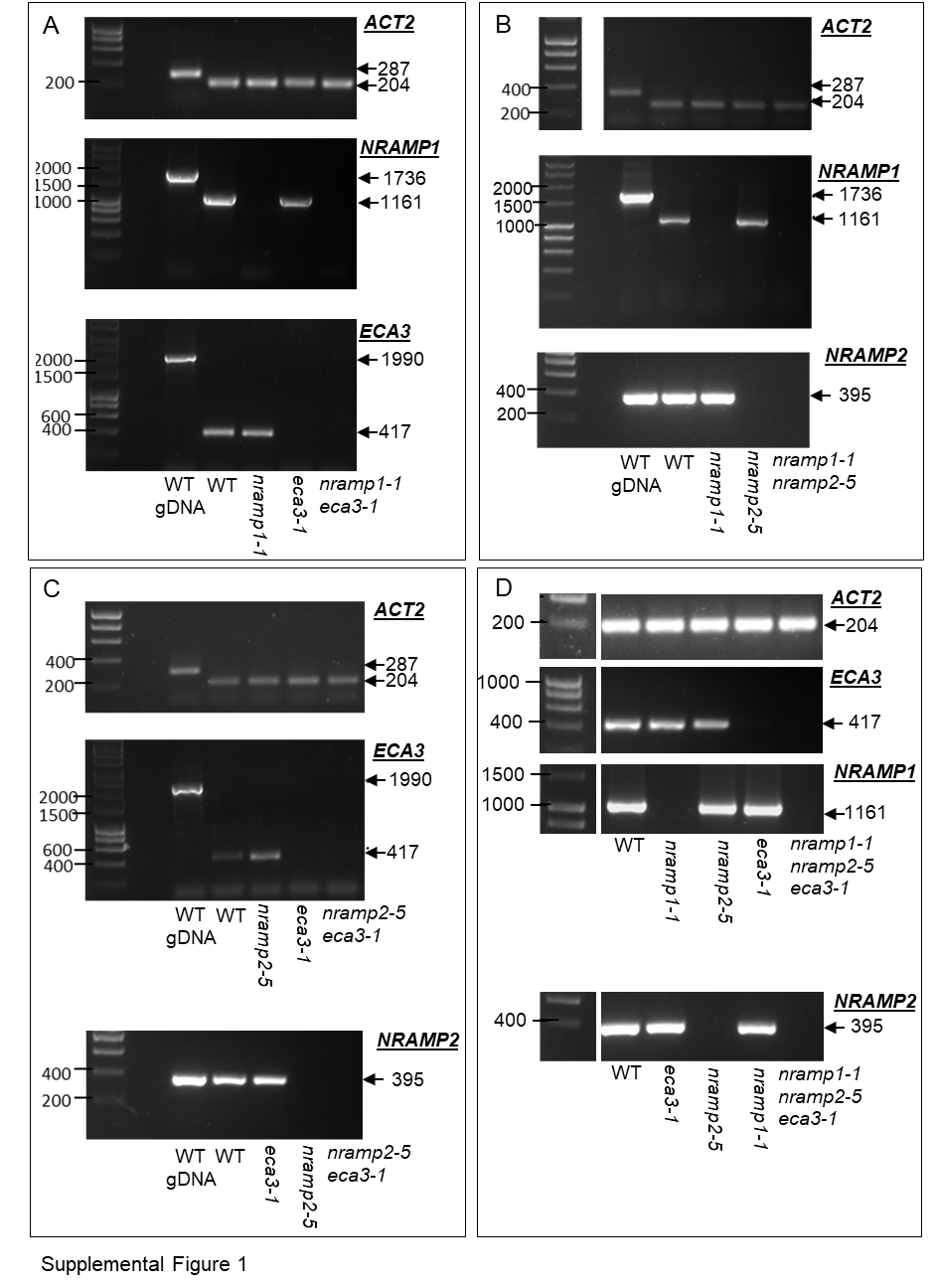
**

**Supplemental Figure 1. Confirmation of *eca* and *nramp* Arabidopsis mutants at RNA level. Related to Figure 1.**

Confirmation of (A) *eca3-1, nramp1-1* and *eca3-1 nramp1-1*, (B) *nramp2-5* and *nramp1-1 nramp2-5*, (C) *eca3-1* *nramp2-5* single and double mutants and (D) the *nramp1-1 nramp2-5 eca3-1* triple mutant. Actin (*ACT2*) is amplified as a control from all samples. *ECA3*, *NRAMP1* and *NRAMP2* products are amplified from WT cDNA samples prepared from reverse transcribed RNA but are absent from relevant single, double and triple mutants. Sizes of molecular weight marker on left; predicted sizes of amplified fragments listed on right of gels.

**
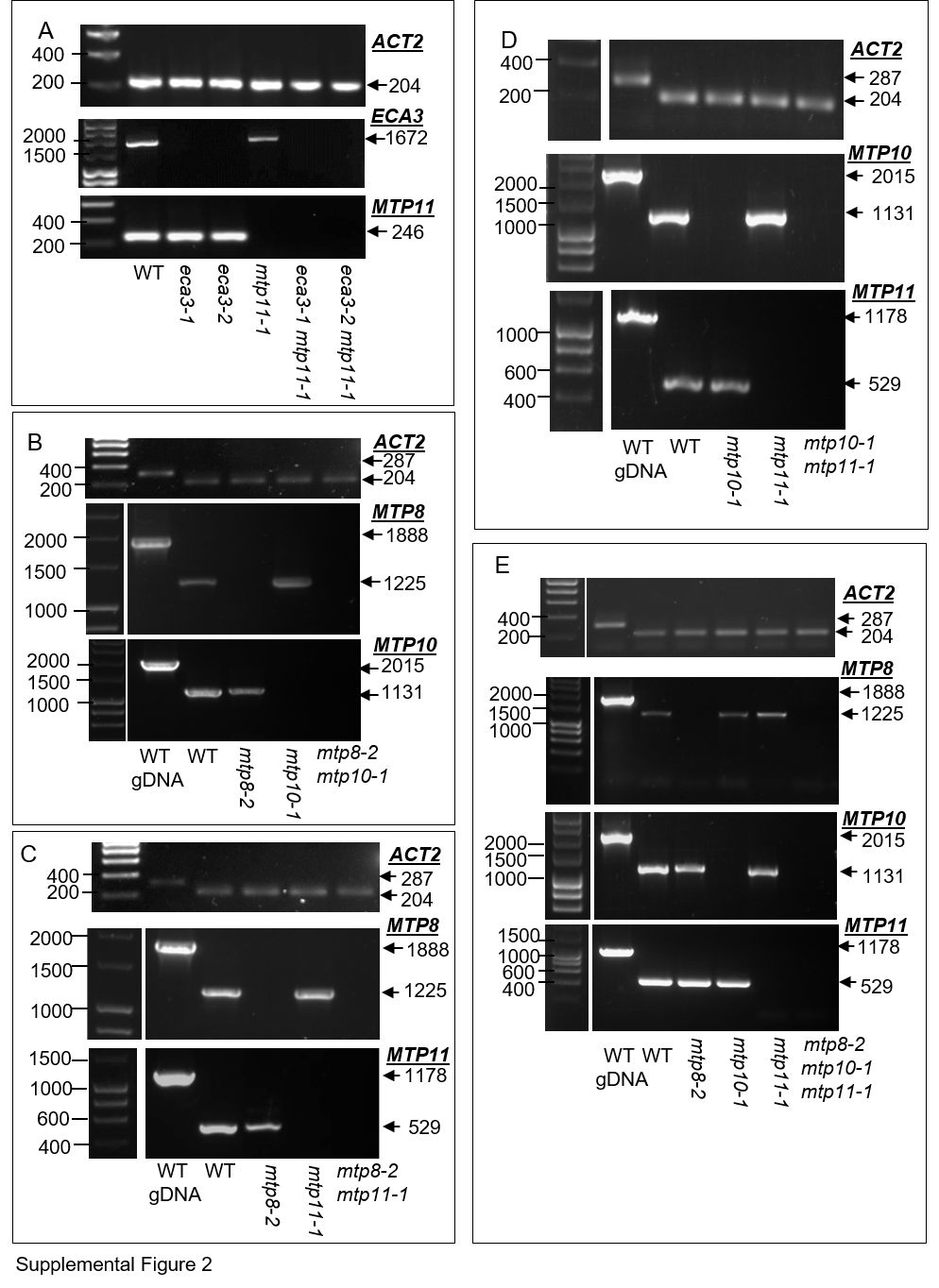
**

**Supplemental Figure 2. Confirmation of *eca* and *mtp* Arabidopsis mutants at RNA level. Related to Figure 2.**

Confirmation of (A) *eca3-1 mtp11-1* and *eca3-2 mtp11-1*, (B) *mtp8-2 mtp10-1*, (C) *mtp8-2 mtp11-1*, (D) *mtp10-1 mtp11-1* double mutants and (E) *mtp8-2 mtp10-1 mtp11-1* triple mutants. Actin (*ACT2*) is amplified as a control from all samples. *ECA3*, *MTP8*, *MTP10* and *MTP11* products are amplified from WT cDNA samples prepared from reverse transcribed RNA but are absent from relevant single, double and triple mutants. Sizes of molecular weight marker on left; predicted sizes of amplified fragments listed on right of gels.


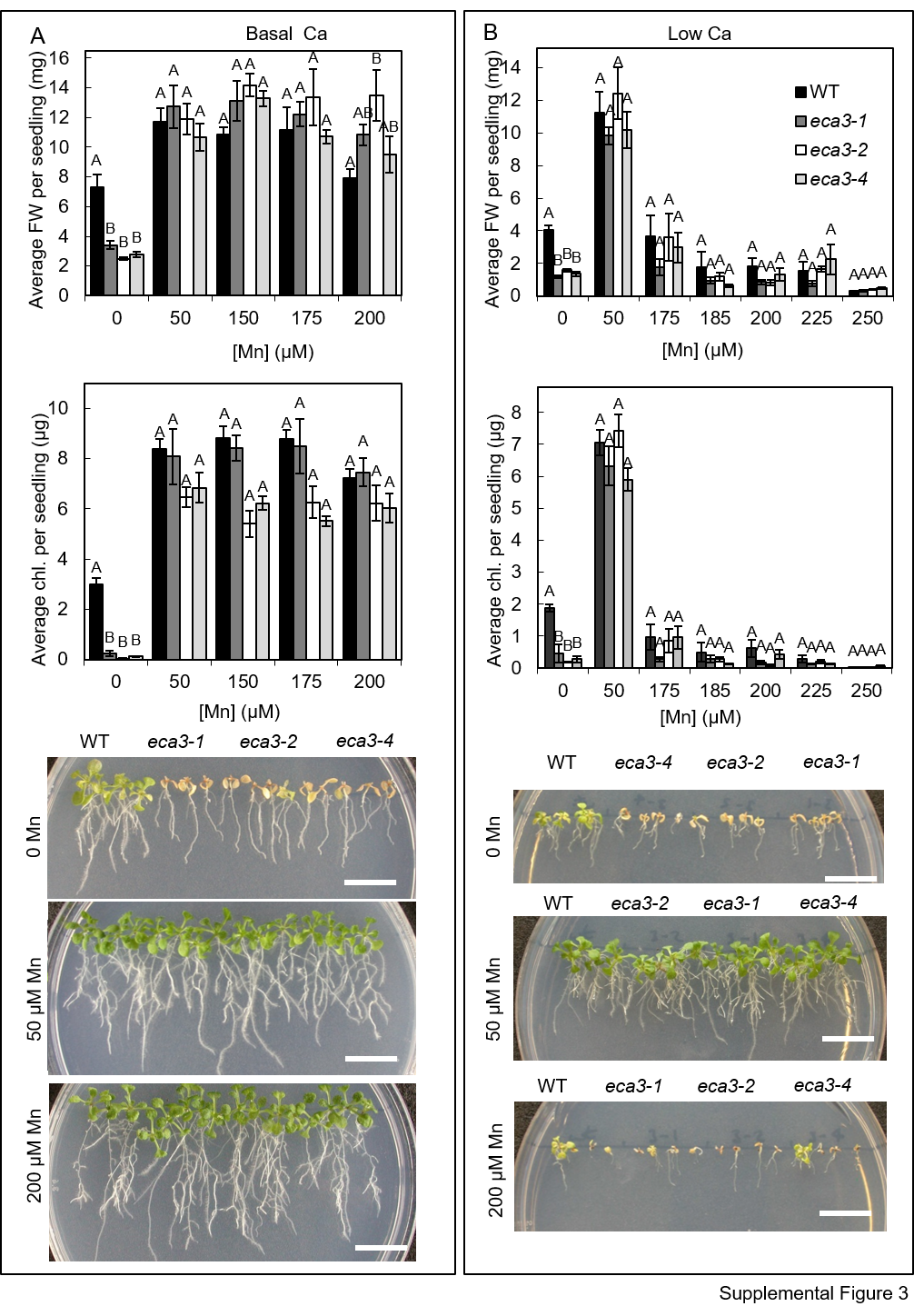


**Supplemental Figure 3. Three *eca3* insertion mutants are sensitive to Mn deficiency, but not to Mn toxicity compared to WT. Related to Figure 2.**

Average fresh weight (FW; mg) and average chlorophyll (Chl; µg) per seedling of Col8 WT, *eca3-1, eca3-2* and *eca3-4* mutants when grown for 20 days on ½ MS supplemented with a range of MnSO_4_ concentrations, and either (A) 1495µM CaCl_2_ (basal Ca) or (B) 100 µM CaCl_2_ (low Ca). Data shows mean FW (mg) per seedling (+SE) calculated for 6 plates, with 4 seedlings per genotype per plate. Statistical significance was assessed with two-way ANOVA and Tukey *post-hoc* test. Means not sharing a letter at a particular condition are significantly different. Photographs display representative growth under different Mn conditions. White bar = 1 cm.


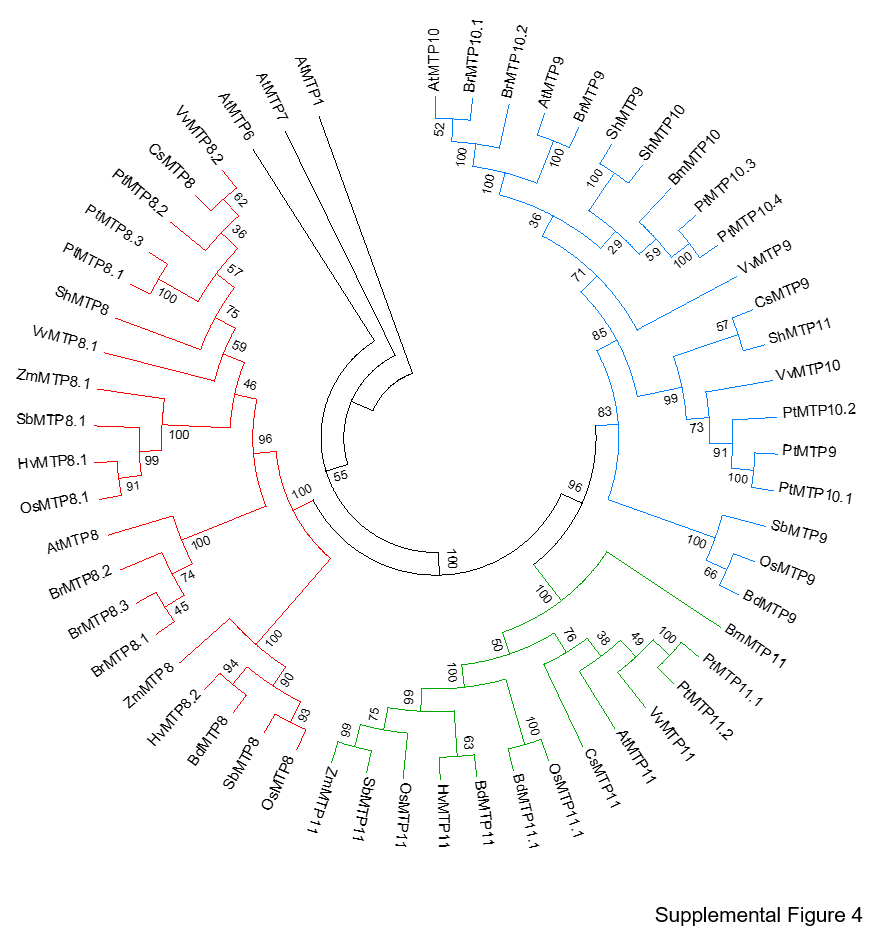


**Supplemental Figure 4. Evolutionary relationship of putative Mn-MTPs from different plant species shows clustering into 3 main sub-clades. Related to Figure 4 and Supplemental Table 2.**

Red, Group 8 MTP8 and MTP8.1; green, Group 9 MTP11 and MTP11.1; blue, Group 9 MTP9 and MTP10. Arabidopsis MTP1, MTP6 and MTP7 included as controls for other MTP sub groups. Evolutionary relationships inferred using the Neighbour-Joining method; bootstrap consensus inferred from 1000 replicates and is taken to represent the evolutionary history of the proteins analysed. The evolutionary distances were computed using the Poisson correction method and are in the units of the number of amino acid substitutions per site. The analysis involved 56 amino acid sequences. All ambiguous positions were removed for each sequence pair. Evolutionary analyses were conducted in MEGA7 phylogenetic analysis package (Kumar et al., 2016). Protein sequences obtained from: *Arabidopsis thaliana* (At), *Brassica rapa* (Br), *Beta vulgaris* spp. maritima (Bm), *Populus trichocarpa* (Pt), *Sorghum bicolor* (Sb), *Brachypodium distachyon* (Bd), *Cucumbis sativis* (Cs), *Oryza sativa* (Os), *Zea mays* (Zm), *Hordeum vulgare* (Hv), *Vitis vinifera* (Vv), *Stylosanthes hamata* (Sh).


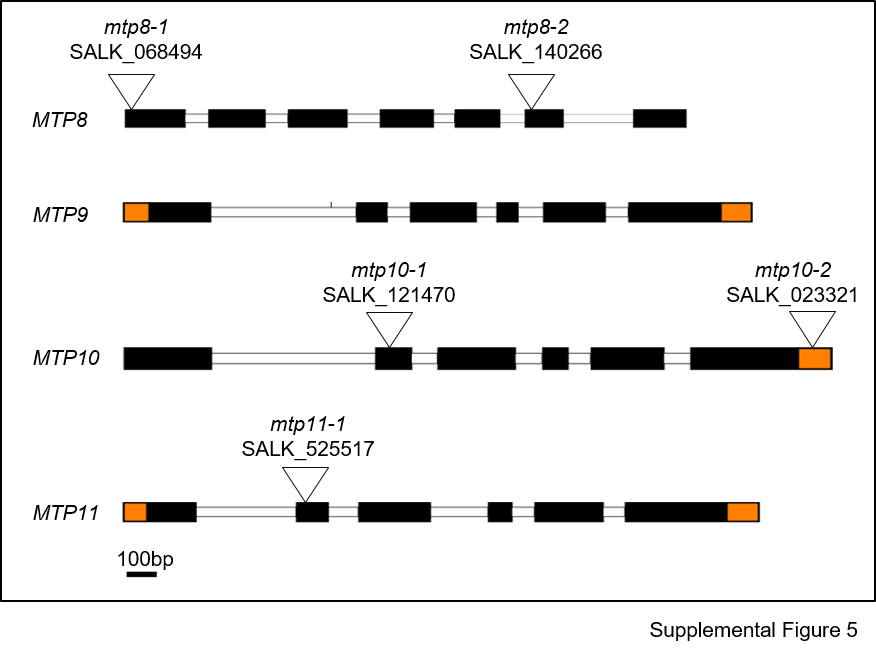


**Supplemental Figure 5. Insertion sites for T-DNA mutants for *MTP8, MTP10* and *MTP11*, confirmed by sequencing, labelled on genomic schematic. Schematic for *MTP9* genomic is included for comparison. Related to Figure 4 and 6.**

Black box, exon; white box, intron; orange box, 5ʹ and 3 ʹ untranslated regions, obtained from TAIR. Arrow, insertion site for *mtp* mutant confirmed by sequencing.


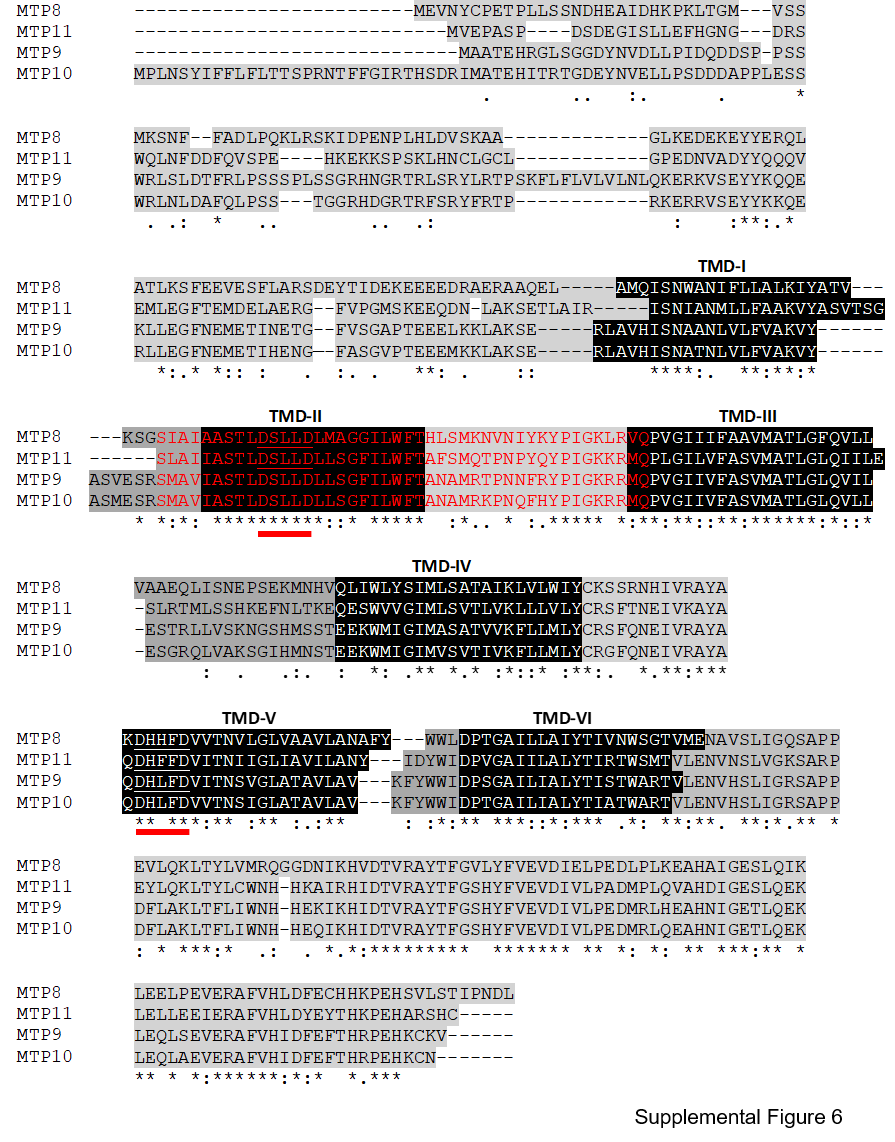


**Supplemental Figure 6. Multiple sequence alignment of MTP8, MTP9, MTP10 and MTP11. Related to Figure 4 and 6.**

Adapted from that generated by ClustalOmega (Seivers et al., 2011). (*) fully conserved residues between sequences; (:) conservation of residues with strongly similar properties; (.) conservation of residues with weakly similar properties. Transmembrane domains (TMDs; highlighted in black) predicted by AramTmConsens (Schwacke et al., 2003). CDF signature sequence marked by red letters. DxxxD domains of Mn-CDFs underlined in red at TMDs 2 and 5.


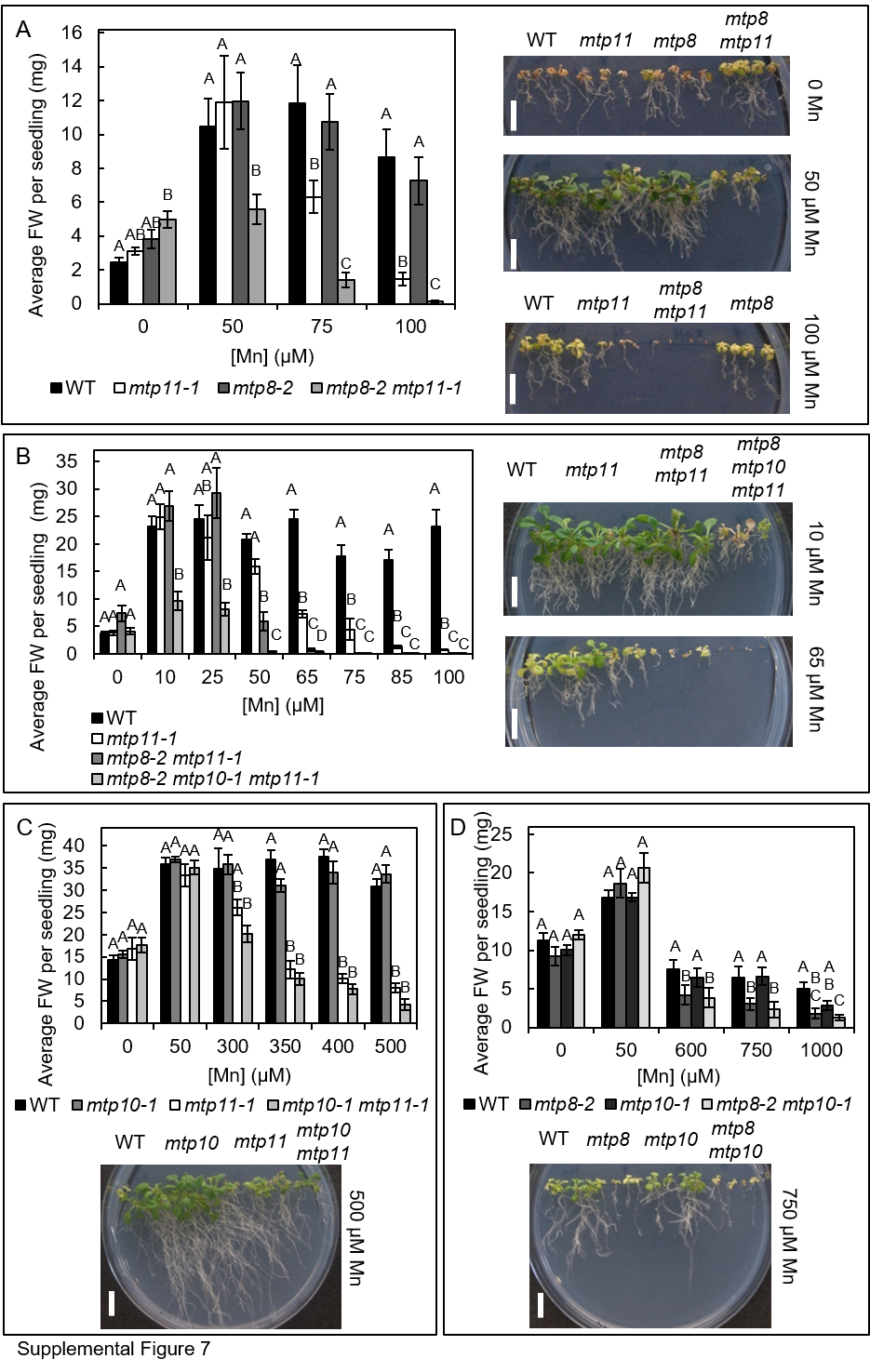


**Supplemental Figure 7. Increased susceptibility of *mtp8 mtp11* and *mtp8 mtp10 mtp11* under low Ca conditions. Related to Figure 4 and 6.**

Comparison of Col8, WT and *mtp11-1* with either *mtp8-2* and *mtp8-2 mtp11-1* (A) or *mtp8-2 mtp11-1* and *mtp8-2 mtp10-1 mtp11-1* (B) under Mn toxicity, low Ca conditions. Plants were grown for 24 days on ½ MS supplied with 100 µM CaCl_2_ and a range of MnSO_4_ concentrations. The *mtp10-1 mtp11-1* (C) and *mtp8-2 mtp10-1* (D) double mutants do not show increases susceptibility to Mn toxicity when compared to WT and single mutants, under basal Ca conditions. Plants were grown for 21 days on ½ MS supplied with 1495 µM CaCl_2_ and a range of MnSO_4_ concentrations. Data shows mean fresh weight (FW; mg) calculated for 6 plates (+SE) with 4 seedlings per genotype per plate. Statistical significance was assessed with two-way ANOVA and Tukey *post-hoc* test. Means not sharing a letter at a particular condition are significantly different. Photographs display representative growth under different Mn conditions. White bar = 1 cm.


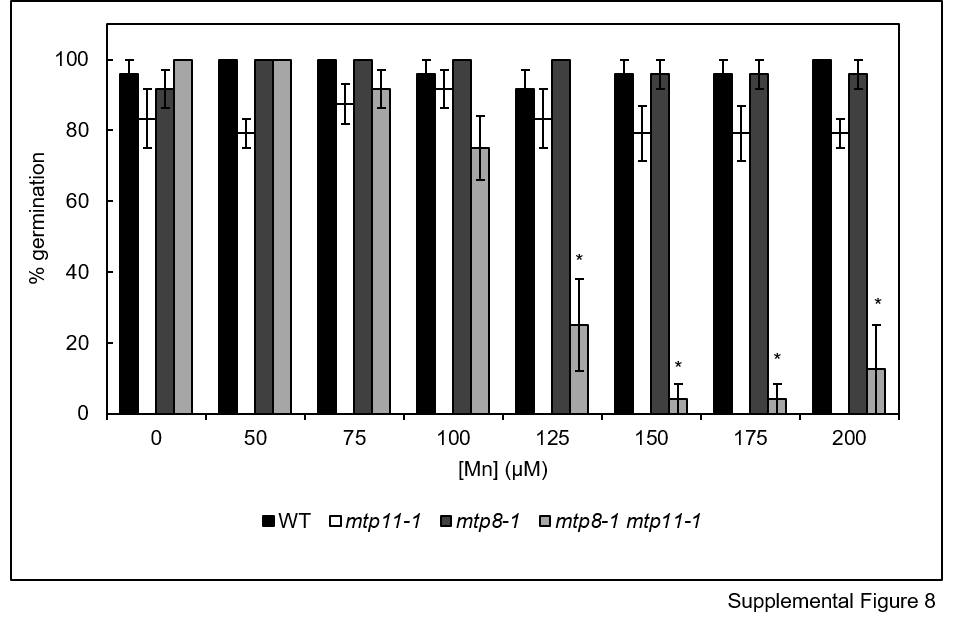


**Supplemental Figure 8. Germination rate of *mtp8-2 mtp11-1* decreases at high Mn concentrations. Related to Figure 4 and 6.**

Comparison of Col8, WT, *mtp11-1* and *mtp8-2 mtp11-1* germination rates under Mn toxicity, low Ca conditions. Plants were grown for 24 days on ½ MS supplied with 100 µM CaCl_2_ and a range of MnSO_4_ concentrations. *mtp8-2 mtp11-1* mutants show decreased germination rates at high Mn concentrations. Data shows mean fresh weight (FW; mg) calculated for 6 plates (+SE) with 4 seedlings per genotype per plate. Statistical significance was assessed with two-way ANOVA and Tukey *post-hoc* test. *= Means are significantly different to WT.


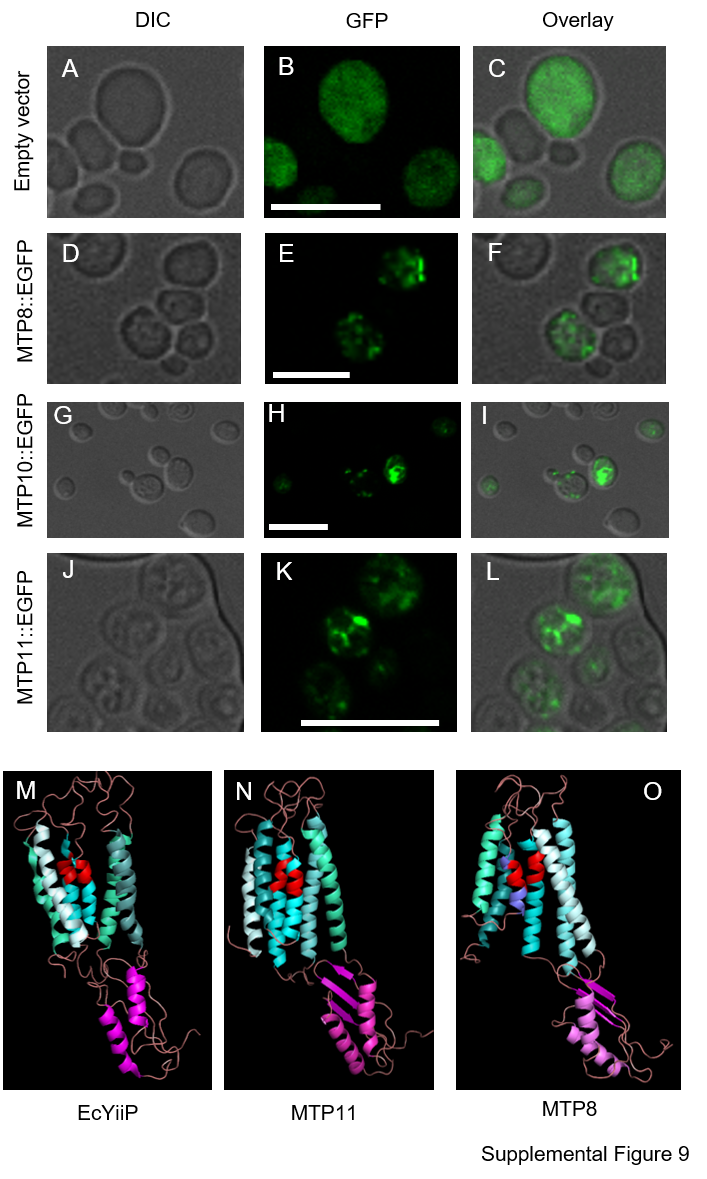


**Supplemental Figure 9. MTP8, MTP10 and MTP11 target intracellular membranes when expressed in yeast. Hypothetical tertiary structure of MTP8, MTP11 and EcYiiP. Related to Figure 9.**

Differential interference contrast (DIC), GFP fluorescence and overlay of DIC and fluorescence of BY4741 yeast transformed with (A-C) empty pAG426galEGFP vector, (D-F) PGAL::MTP8::EGFP, (G-I) PGAL::MTP10::EGFP and (J-L) PGAL::MTP11::EGFP. White scale bar = 5 µm. (M-O) Hypothetical tertiary structure of MTP8 and MTP11, compared with EcYiiP. Based on homology model using EcYiiP as template. Model constructed and visualised using Swiss-Model (Biasini et al., 2014) and The PyMOL Molecular Graphics System, Version 1.8 Schrödinger, LLC.

**Supplemental Movie 1. MTP8 targets the tonoplast when stably expressed in Arabidopsis. Related to Figure 6 and 7.**

Timelapse movie of P35S::MTP8::GFP (green signal) expressed stably in Arabidopsis and imaged after 5 days of growth on ½ MS. Movie shows formation of transvacuolar strands (TVS) and movement of TVS across cell. Interval = 1.4 seconds

| Supplemental Table 1. Primers used in this study to confirm zygosity of single, double and triple insertion mutants and to amplify coding sequences for cloning. | | |
| --- | --- | --- |
| Primer | **Target** | **Sequence** |
| Targeting T-DNA | | |
| LBa1 | pROC2 | 5ʹ-GCGTGGACCGCTTGCTGCAACT |
| GABI_LB | pAC106 | 5ʹ-ATATTGACCATCATACTCATTGC |
| Targeting WT gene of interest for genotyping | | |
| MTP8F | *MTP8* | 5ʹ-TGGTTGCGGCCGTTCTTGCTA |
| MTP82R | *MTP8* | 5ʹ-AGGACGGAATGTTCAGGCTTGTGA |
| MTP8_topowithstop | *MTP8* | 5ʹ-TCATAAATCGTTGGGGATTGTA |
| MTP10F | *MTP10* | 5ʹ-GATGCGCCGCCGCTTGAATC |
| MTP10R2 | *MTP10* | 5ʹ-GAGACGGCACAACGTTCAAGTATCT |
| MTP11seq43.F | *MTP11* | 5ʹ-CTGCTCGAGTTTCACGGTAAC |
| MTP11jkp1.R  MTP11seq101.F  MTP11ex3.R  ECA3ex10F  ECA3SGR | *MTP11*  *MTP11*  *MTP11*  *ECA3*  *ECA3* | 5ʹ-AATCTGCAATCCAAGTGTTGC  5ʹ-AGGTTTCACCGGAACACAAG  5ʹ-GAAGCATGTTTGCAATGTTTG  5ʹ-CATACACGATTCTATGTTGCAGACAGATGAT  5ʹ-GTTACCAAATTGACCCACAGAAGT |
| NRAMP1b_F | *NRAMP1* | 5ʹ-AGGCCCTGGTTTTCTTGTTT |
| NRAMP1R | *NRAMP1* | 5ʹ-GGAACCAACGCAAACGGGAGCT |
| NRAMP2_promF | *NRAMP2* | 5ʹ-TTCAAAATTCAGGTACGTCGAC |
| NRAMP2R | *NRAMP2* | 5ʹ-AGCTTCTAACTTCCTCACACCG |
| Actin2F | *ACT2* | 5ʹ-GGTAACATTGTGCTCAGTGGTGG |
| Actin2R | *ACT2* | 5ʹ-CTCGGCCTTGGAGATCCACATC |
| Targeting WT gene of interest for amplification for cloning | | |
| MTP82F | *MTP8* | 5ʹ-CACCATGGAAGTCAATTATTGTCC |
| MTP8topoF | *MTP8* | 5ʹ-TTATTGTCCGGAAACACCGTT |
| MTP8topowithstop | *MTP8* | 5ʹ-TCATAAATCGTTGGGGATTGTA |
| MTP8toponostop | *MTP8* | 5ʹ-TAAATCGTTGGGGATTGTA |
| MTP9_topoF | *MTP9* | 5ʹ-CACCATGGCGGCGACGGAGCAT |
| MTP9_topowithstop | *MTP9* | 5ʹ-TCAAACCTTGCATTTGTGTTCTG |
| MTP9_toponostop | *MTP9* | 5ʹ-AACCTTGCATTTGTGTTCTG |
| MTP10_topoF | *MTP10* | 5ʹ-CACCATGCCGCTTAACTCCTA |
| MTP10_topowithstop | *MTP10* | 5ʹ-CTAGTTACACTTGTGTTCAGGACGA |
| MTPP10_toponostop | *MTP10* | 5ʹ-GTTACACTTGTGTTCAGGACGATGA |
| MTP11_topoF | *MTP11* | 5ʹ-CACCATGGTTGAGCCAGC |
| MTP11_topowithstop | *MTP11* | 5ʹ-CTAACAGTGGGATCTAGCGTGC |
| MTP11_toponostop | *MTP11* | 5ʹ-ACAGTGGGATCTAGCGTGC |
| Generating site-directed mutations | | |
| MTP8_D258H_F | *MTP8* | 5'-CCACATCAAAGTGATGATGCTTTGCATATGCACGGAC |
| MTP8_D258H_R | *MTP8* | 5'-GTCCGTGCATATGCAAAGCATCATCACTTTGATGTGG |
| MTP11_D249H_F | *MTP11* | 5'-GAGATCGTTAAAGCTTATGCTCAACATCATTTCTTCGACG |
| MTP11_D249H_R | *MTP11* | 5'-GATGAAGCCAGAAAGAAGAGCAAGAAGAGAGTCCAATGT |
| Determining expression of *MTP8* and *MTP10* in OE Arabidopsis lines | | |
| AtMTP8rtF1 | *MTP8* | 5'-GGACAATCAGCTCCTCCAGA |
| AtMTP8rtR1 | *MTP8* | 5'-GAAGGTATATGCACGGACGG |
| AtMTP10rtF2 | *MTP10* | 5'-ACGTCCATTCACTGATCGGC |
| AtMTP10rtR2 | *MTP10* | 5'-GTGACCCAAAAGTGTAAGCCC |
| AtUBQ10rtF | *UBQ10* | 5'-CGTCTTCGTGGTGGTTTCTAA |
| AtUBQ10rtR | *UBQ10* | 5'-GGATTATACAAGGCCCCAAAA |
